# Chronic Wasting Disease: Transmission Mechanisms and the Possibility of Harvest Management

**DOI:** 10.1371/journal.pone.0151039

**Published:** 2016-03-10

**Authors:** Alex Potapov, Evelyn Merrill, Margo Pybus, Mark A. Lewis

**Affiliations:** 1 Department of Biological Sciences, University of Alberta, Edmonton, Alberta, Canada; 2 Centre for Mathematical Biology, University of Alberta, Edmonton, Alberta, Canada; 3 Department of Mathematical and Statistical Sciences, University of Alberta, Edmonton, Alberta, Canada; 4 Alberta Sustainable Resource Development, Edmonton, Alberta, Canada; Colorado State University, College of Veterinary Medicine and Biomedical Sciences, UNITED STATES

## Abstract

We develop a model of CWD management by nonselective deer harvest, currently the most feasible approach available for managing CWD in wild populations. We use the model to explore the effects of 6 common harvest strategies on disease prevalence and to identify potential optimal harvest policies for reducing disease prevalence without population collapse. The model includes 4 deer categories (juveniles, adult females, younger adult males, older adult males) that may be harvested at different rates, a food-based carrying capacity, which influences juvenile survival but not adult reproduction or survival, and seasonal force of infection terms for each deer category under differing frequency-dependent transmission dynamics resulting from environmental and direct contact mechanisms. Numerical experiments show that the interval of transmission coefficients β where the disease can be controlled is generally narrow and efficiency of a harvest policy to reduce disease prevalence depends crucially on the details of the disease transmission mechanism, in particular on the intensity of disease transmission to juveniles and the potential differences in the behavior of older and younger males that influence contact rates. Optimal harvest policy to minimize disease prevalence for each of the assumed transmission mechanisms is shown to depend on harvest intensity. Across mechanisms, a harvest that focuses on antlered deer, without distinguishing between age classes reduces disease prevalence most consistently, whereas distinguishing between young and older antlered deer produces higher uncertainty in the harvest effects on disease prevalence. Our results show that, despite uncertainties, a modelling approach can determine classes of harvest strategy that are most likely to be effective in combatting CWD.

## 1. Introduction

CWD is a transmissible spongiform encephalopathy (TSE), like mad cow disease (or BSE) in cattle and scrapie in sheep. It is found in cervids, including white-tailed deer (*Odocoileus virginianus*), mule deer (*O*. *hemionus*), elk (*Cervus canadensis*), and moose (*Alces alces*) [1,2]. To date it has been detected in over seventeen US states and two Canadian provinces. Herd reduction by hunting or government culling programs is the most common CWD management tool because there is no current vaccine or treatment. During a major portion of the disease progression, infected and uninfected individuals cannot be distinguished visually, so any population harvest has to be primarily nonselective with respect to infection status. Attempts to model the effect of such management actions show that their efficiency may depend critically on the mechanisms of disease spread [3]. In particular, if the transmission mechanisms are density-dependent (DD), herd reduction may have the potential to decrease prevalence, while if frequency-dependent (FD), then modeling shows little effect of density reduction [3].

Routes of CWD transmission in wild cervids are not well understood. Transmission is complicated by the properties of CWD prions in the environment and by deer behavior and physiology, as well as by the extent of establishment or progression of disease within local populations. As a result there is insufficient evidence to point to a single, most important mechanism for transmission. Nonetheless, patterns in hunter harvested data indicate that infection rates are higher in males and increase with age, at least up to 5–6 years old [4,5]. Potapov et al. [6] attempted to identify the most plausible transmission routes by comparing modeling outcomes of several major mechanisms to an observed disease occurrence that is two times higher in adult males than females (e.g. [4,5]). Their results supported several possible transmission paths, but generally the most probable mechanisms were FD, including higher transmission to males than other deer due to differences in food consumption, contact rates, or susceptibility; transmission from females to males during mating behaviors; or some related yet unknown reason. Potapov et al. [6] also found seasonal segregation by the sexes to be important, indicating this driving mechanism may be related to disease transmission within social groups. However, more empirical studies are required to determine which paths play the major role in natural CWD transmission.

The purpose of the current paper is to address (1) whether the effect of harvest strategy or policy (i.e. intensities of harvesting different deer categories) for controlling CWD should depend on the type of disease transmission and (2) to identify the optimal harvest strategies for a given transmission mechanism, that is the policy giving the least possible disease prevalence without population collapse. We consider six plausible CWD transmission mechanisms from [6], all of which are FD. Potapov et al. [7] show that herd control under FD transmission by nonselective harvest is possible in species with density-dependent recruitment, such as cervids, because it reduces the lifetime of infected individuals and increases the recruitment of new healthy adults [7]. We start by comparing the effect of commonly used harvest strategies that target different sex and age categories on eradicating and/or reducing prevalence across these transmission routes. In particular, we evaluate harvests targeting young and old males because prevalence is highest in males [4,8,9] and Jennelle et al. [10] found under their modeling assumptions that harvest focused males can result in stable population dynamics and control of CWD within the next 50 years. We then consider optimal harvest, defined as the harvest achieving the lowest disease prevalence without driving the population to extinction, for each transmission mechanism.

In devising optimal harvests under FD transmission, there are several considerations. First, a common goal in harvest management is targeting a population’s density itself. However, this is not an appropriate goal if we assume there is no relationship between deer density and disease prevalence (FD) because population age and sex structures may be different at identical densities. Instead, the FD transmission function depends on ratios in herd composition structure because the different age and sex groups exhibit differential responses to the disease. To simplify our presentation of FD model results, we emphasize model outcomes in terms of proportion of the most harvested deer category removed every year. Second, the full effect of a harvest policy may become evident only after a long time (e.g., 10–50 years, [3,10]). As a result we consider prevalence in the deer population at equilibrium corresponding to the given level of harvest and the harvest policy chosen. The advantage of focusing on the equilibrium is that it is independent of the initial state of both population and the disease (provided there is only one stable steady state). Further, we employ two approaches to finding optimal harvest policies. First we consider harvest effects on disease prevalence that emerge from a combination of harvest regulations and hunter preferences, and then evaluate the robustness of these effects for different levels of total harvest. Second we choose harvest intensity and look for the optimal harvest policy. Here we present results from both approaches.

An understanding of the optimal proportions of age and sex classes in the harvest in relation to the intensity of harvest and the effects on transmission of CWD is important for managers trying to effectively control the disease with the lowest costs, while retaining the public support essential for implementing such actions. From an ecological perspective, the most interesting finding is how harvest affects the disease transmission through a chain of cause and effect. Harvest changes population proportions both directly and through density-dependent juvenile survival. This in turn may change the modes of disease transmission, and eventually may lead to prevalence reduction and even the disease eradication. These effects potentially may be used for management of other wildlife diseases.

In this paper, we first describe the basic features of the model in Section 2. Section 3 presents the major results of our work: the effect of harvest on the disease prevalence under different harvest policies and harvest intensities. In Section 4 we discuss the results and major assumptions. Section 5 contains conclusions.

## 2. Model of Deer Population

We use the model developed in [6] with a few modifications related to male age classes, rates of disease-related mortality and reproduction at low buck:doe ratios that may be important for modeling deer harvest management. The complete model equations are given in Appendix A in S1 File, with further details in [6]. Here we briefly describe the basic model features.

### 2.1. Deer categories

The model has four deer categories: young adult males (*m*1), older adult males (*m*2), adult females (*f*), and juveniles (*j*); the latter are assumed to have a 50:50 sex ratio at birth. Two classes of adult males is a realistic management approach often applied to hunting regulations that permit trophy hunts or to protect older, breeding males in the population. Older male deer (>3.5 yr) typically have a higher number of antler points [11] and thus can be differentiated from younger males (>1.5 but <3.5 yrs) by hunters/managers. We consider two disease stages, susceptible (*S*) and infected (*I*) deer, but omit an incubation stage. Adding incubation stage does not change the model outcomes, as was found in [6]. Therefore, the model has eight compartments: four densities of susceptible deer (*S*_*j*_, *S*_*f*_, *S*_*m1*_, *S*_*m2*_) and four densities of infected, (*I*_*j*_, *I*_*f*_, *I*_*m1*_, *I*_*m2*_). For each deer class we denote the total density by *D*_*x*_ = *S*_*x*_ + *I*_*x*_, where *x* = *j*,*f*,*m*1,*m*2, and the total density for all deer is *D* = *D*_*j*_ + *D*_*f*_ +*D*_*m1*_ + *D*_*m2*_.

### 2.2. Population dynamics

The model includes rates of birth, natural mortality, harvest, and disease transmission. In this model, juveniles with 50:50 sex ratio are produced equally by infected and uninfected females. Juveniles become adults after 1.5 years (in reality they are adults after one year, but most deer reproduce after two years) and young adult males become older males at 3.5 years. All categories of deer are harvested. Harvest rate is set by deer category, but does not depend on infection status, so harvest is nonselective. Susceptible deer become infected at the rate proportional to force of infection λ, which also depends on the deer category. Vertical transmission is present in the model, but was not used in the numerical simulations to simplify the analysis because its effect is small [6] and unlikely [12].

The birth rate is assumed to be density-independent and equal for infected and uninfected females. However, we assume that it decreases at low buck:doe ratio *D*_*m*_ / *D*_*f*_: if there are less than 5 bucks per 100 does, the birth rate linearly decreases with *D*_*m*_ / *D*_*f*_, reaching zero in the absence of males.

### 2.3. Density-dependent juvenile mortality

Per capita mortality rate for adults is assumed to be density-independent [13]. The mortality rate of infected deer is assumed to increase compared to that of uninfected deer by the value μ, which approximately is the inverse mean duration of the disease [14]. We model density-dependence through juvenile mortality, which we assume is related to food limitation. We use the juvenile mortality model developed in [6] based on the ratio of required *F*_*R*_ and available *F*_*A*_ food for the deer population: juvenile mortality is density independent when *F*_*R*_ < *F*_*A*_, and then grows with population starvation when *F*_*R*_ > *F*_*A*_ (see also [15]). The resulting nonlinear per capita juvenile recruitment rates resemble those presented in [16]. The required food is estimated based on daily food consumption rates *F*_*S*,*x*_,*F*_*I*,*x*_, *x* = *j*,*f*,*m* for each deer category (see Tables A1, A2 in Appendix A in S1 File), and the available food is estimated from the deer densities of a healthy equilibrium population, which is assumed to be known. For brevity we refer to the above food-based, density-dependent juvenile mortality model as the “starvation model”. To test the results of disease control against the other forms of density-dependent juvenile mortality, we used two alternative models, which are based upon the expression given in [17] and also uses the ratio of *F*_*R*_ / *F*_*A*_(see details in Appendix A in S1 File).

### 2.4. Disease transmission

In [6] we develop the mathematical expressions for the force of infection terms for 7 transmission mechanisms and the four of them that are considered most plausible. The detailed mathematical expressions for force of infection terms are given in Appendix A in S1 File, here we give only the main assumptions of these expressions.

1. The disease transmission is mainly frequency-dependent, that is transmission occurs primarily within deer groups.
2. There is seasonality in group structure: summer there are separate adult (> 1.5 yrs) male groups and family group (females and young ≤ 1.5 yrs), while in winter there are mixed groups. Contribution of each type of groups to disease transmission is described by seasonal weights *w*_*S*_, *w*_*M*_ for separate and mixed respectively.
3. The magnitude of the force of infection terms is determined by transmission coefficient β, but disease transmission between different deer classes may not be equal. The relative intensity of transmission from class *v* to class *u* is described by the matrix of transmission weights ψ_*uv*_ where *u* and *v* may be equal to *j*, *f*, or *m*. Expressions for the force of infection use weighted sums of infected deer densities.
4. We use the following choices for transmission weights that were identified in [6] as plausible:
  - null hypothesis or equal transmission among all groups ψ_*uv*_ = 1. Usually we equate transmission with direct contacts between deer because information about these contacts is not currently available;
  - food-mediated environmental transmission with ψ_*uv*_ proportional to food consumption by different deer classes. This gives high transmission rate for males, medium rate for females, and low rate for juveniles;
  - higher relative susceptibility of males to infection described by coefficient *Y*_*m*_>1, then ψ_*mv*_ = *Y*_*m*_, all other ψ_*uv*_ = 1;
  - higher relative rate of contacts between males described by coefficient *Z*_*m*_>1, then ψ_*mm*_ = *Z*_*m*_, all other ψ_*uv*_ = 1.
5. An important disease transmission path may be transmission from females to males during rut, and the corresponding term may be added to the force of infection for males. We assume that if there are enough older males, they alone mate with females, but if the density of the older males drops below one older male per more than ten females, that is the ratio *D*_*m*2_ / *D*_*f*_ < 0.1, then younger males also take part in mating. Participation of younger males in mating under lack of older ones is described in the literature [18]; however we did not find quantitative estimates for the threshold.

Table 1 describes six types of disease transmission scenarios based upon the described transmission mechanisms and their combinations used in the calculations below. The scenarios are denoted as **TM1** to **TM6**. Potapov et al. [6] show that all except the pure null model (TM1) were capable of reproducing the observed difference in CWD prevalence between males and females found in many jurisdictions [4,5].

**Table 1:** Transmission mechanisms, fitted transmission coefficients (β and one of *w*_*S*_,β_*R*_,*Y*_*m*_,*Z*_*m*_), and population characteristics at the developed stage of the disease (adult prevalence π_*a*_ and male/female prevalence ratio *r*_*mf*_).

Transmission mechanism	Direct/Indirect transmission and seasonality	Rut transmission	Fitted parameters and developed stage
**TM1**	Equal transmission between all deer classes (ψ_*uv*_ = 1); *w*_*S*_ fitted.	No	β = 0.60 *w*_*S*_ = 1.68 π_*a*_ = 0.41 *r*_*mf*_ = 1.18
**TM2**	Equal transmission between all deer classes (ψ_*uv*_ = 1); *w*_*S*_ = 1	Yes	β = 0.49 β_*R*_ = 0.48 π_*a*_ = 0.30 *r*_*mf*_ = 1.56
**TM3**	Food-mediated transmission (high to males, medium to females, low to juveniles); *w*_*S*_ fitted.	No	β = 0.82 *w*_*S*_ = 1.02 π_*a*_ = 0.23 *r*_*mf*_ = 1.86
**TM4**	Food-mediated transmission (high to males, medium to females, low to juveniles); *w*_*S*_ = 1	Yes	β = 0.80 β_*R*_ = 0.04 π_*a*_ = 0.23 *r*_*mf*_ = 1.90
**TM5**	Equal transmission between all deer classes, but male susceptibility is higher (*Y*_*m*_>1); *w*_*S*_ = 1	No	β = 0.49 *Y*_*m*_ = 1.56 π_*a*_ = 0.32 *r*_*mf*_ = 1.58
**TM6**	Increased male-to-male transmission (*Z*_*m*_>1), equal transmission between other deer classes; *w*_*S*_ = 1.	No	β = 0.49 *Z*_*m*_ = 1.66 π_*a*_ = 0.31 *r*_*mf*_ = 1.55

We do not explicitly model an environmental compartment, where prions may potentially accumulate. As we have shown in [6], if prions become practically inaccessible to deer after 1–2 years, as is likely, the concentration of accessible prions in the environment is simply proportional to the local density of infected deer. Mathematically this leads to a model having the same form as one for direct transmission. In six transmission mechanisms mentioned above, this approach is implemented as food-mediated environmental transmission. See [6] for more details.

### 2.5 Disease-related mortality: non-exponential distribution of lifetime

According to the literature, CWD-infected deer live about 2 years [19]. The exact duration varies, but values that are much smaller or much larger values have not been reported in the literature. At the same time, simple one-compartment mortality models of the type $\dot{u}=-\mu u$ give an exponential distribution of lifetimes since the population size decreases according to *u*(*t*) = *u*_0_ exp(−μ*t*). If μ = 0.57year^−1^ [14], then the mean duration of lifetime is 21 month, although 90% of individuals die between 1 and 68 months, that is, variability is very high. This distribution can be changed by splitting the infected stage into many compartments with quick enough transition between them to keep the mean duration of the infected stage the same (see e.g., [20]). The population within each compartment is described by Gamma distribution, and, as the number of compartments increases, the sum of these distributions is approaching a step-like function with diminishing variety in survival times. For 5 compartments 90% individuals die between 8 and 38 months, and for 25 compartments–between 15 and 28 months. In this paper we present results for *n*_*c*_ = 25 compartments (some other cases are considered in Appendix A in S1 File). For natural deer mortality we still use a single compartment per deer category and exponential distribution of lifetimes.

### 2.6. Hunter harvest

Harvest in our model is described by four per capita harvest rates *h*_*j*_,*h*_*f*_,*h*_*m*1_,*h*_*m*2_. We assume they may be different for each deer category but are equal for infected and uninfected individuals. Nonetheless, we evaluate the sensitivity of the latter assumption because the behavior of infected deer itself may still make them more or less susceptible to harvest. We refer to the maximum of these partial rates *h* = max{*h*_*j*_, *h*_*f*_, *h*_*m*1_, *h*_*m*2_} as the **harvest intensity**, and the set of relative rates *h*_*Px*_ = *h*_*x*_ / *h*, *x* = *j*,*f*,*m*1,*m*2 as the **harvest preferences**. The set of harvest preferences **h**_*P*_ = (*h*_*Pj*_, *h*_*Pf*_, *h*_*Pm*1_, *h*_*Pm*2_) is referred to as the **harvest policy** or **strategy**. The most intensively harvested deer category always has *h*_*Px*_ = 1. In a general season, typically the preference is for males of both categories. We assume that the harvest intensity *h* is primarily related to the total number of licenses while *h*_*Px*_ reflect hunter preferences when they buy a general license or are limited by imposed license restrictions. In the latter case the preferences can be determined from comparison of proportions of different types of deer in the wild population and in the hunter harvest, see [6].

Harvest intensity is modeled by an instant rate of removal of individuals, while managers typically characterize harvest by the number of individuals or proportion of species/sex/age group harvested annually. For this reason we represent the results in terms of the proportion *H* of the most intensely harvested deer category *x* removed annually, that is the one for which *h*_*Px*_ = 1. At equilibrium *H* is related to the harvest intensity *h* as *H* = *h*/(1+*h*) provided *h* is in units year^−1^, see Appendix A in S1 File for mathematical details.

Below we use seven harvest policies **h**_*P*_ = (*h*_*Pj*_, *h*_*Pf*_, *h*_*Pm*1_, *h*_*Pm*2_) that arise from common regulations and hunter preferences, see Table 2. The first two policies are related to general seasons where harvests are unrestricted, but hunters show preferences for what they harvest. Preferences were derived from actual data and were estimated by comparing proportions of the categories in the population and in the harvest records from Wildlife Management Units (WMUs) with mandatory head submission policy in eastern Alberta and at Canadian Forces Base Wainwright (CFBW). The other five harvest regulations are related to restricted harvest of deer categories. We evaluate these policies under a range of harvest intensities *h*.

**Table 2:** Harvest preferences used in calculations in Fig 3.

#	Type of harvest policy	Values of harvest preferences(*h*_*Pj*_, *h*_*Pf*_, *h*_*Pm*1_, *h*_*Pm*2_)
HP1	General season, eastern Alberta	0.23, 0.41, 1, 1
HP2	General season, Wainwright Canada Forces Base (CFBW)	0.23, 0.33, 1, 1
HP3	Antlered deer (all adult males) only	0, 0, 1, 1
HP4	Young antlered deer only	0, 0, 1, 0
HP5	Older antlered deer only, also called trophy or quality deer management (Jenks et al 2002)	0, 0, 0, 1
HP6	Antlerless deer only	1, 1, 0, 0
HP7	Non-preferred harvest or culling	1, 1, 1, 1

Besides these seven strategies, we find the policy **h**_*P*_ that gives the least disease prevalence for a given *h*. We call this policy an optimal policy since this is a result of prevalence optimization. We consider two types of optimal policy: a) where only antlerless and antlered harvest are chosen independently so *h*_*Pm*1_ = *h*_*Pm*2_; and b) where three adult preferences *h*_*Pf*_, *h*_*Pm*1,_ *h*_*Pm*2_ are chosen independently. Adding juvenile preference as a separate control appears to be excessive, and we use *h*_*Pj*_ = 0.6*h*_*Pf*_, the latter relation is taken from eastern Alberta harvest policy.

### 2.7. Model parameterization

Deer population data were taken from [6] and given in Table A2 in S1 File. The disease transmission parameters were obtained by fitting our model with Eastern Alberta harvest policy and intensity to Alberta CWD prevalence data for hunter harvested deer in 2006–2012 [21]. Due to a small amount of data, only 1 or 2 transmission parameters could effectively be fitted, the fitted parameters are listed in Table 1. The greater number of fitted parameters is not supported by AIC criterion as well.

For maximum likelihood fitting, we assumed that the number of infected deer harvested by hunters can be modelled by Poisson random variable with a given intensity. The intensity parameter of the Poisson distribution is proportional to CWD prevalence in males and females, as given by the model.

## 3. Results

### 3.1 Disease transmission parameters and predicted CWD prevalence

The result of fitting of the transmission parameters for transmission mechanisms TM1-TM6 are shown in Table 1. Besides the values of the fitted transmission parameters, there are two parameters characterizing the disease at the developed stage, when the density and the prevalence stabilize: adult disease prevalence π_*a*_ and male to female prevalence ratio *r*_*mf*_. The following features can be seen:

- The highest disease prevalence arises for the mechanisms TM1;
- The highest male to female prevalence ratio of 1.8 arises for TM3;
- The results for TM4 appear to be the same as for TM3 because the fitted value of rut transmission coefficient is close to zero, so below we merge the two models and denote it as TM3,4;

### 3.2 Harvest intensity and harvest policy

**Fig 1:**
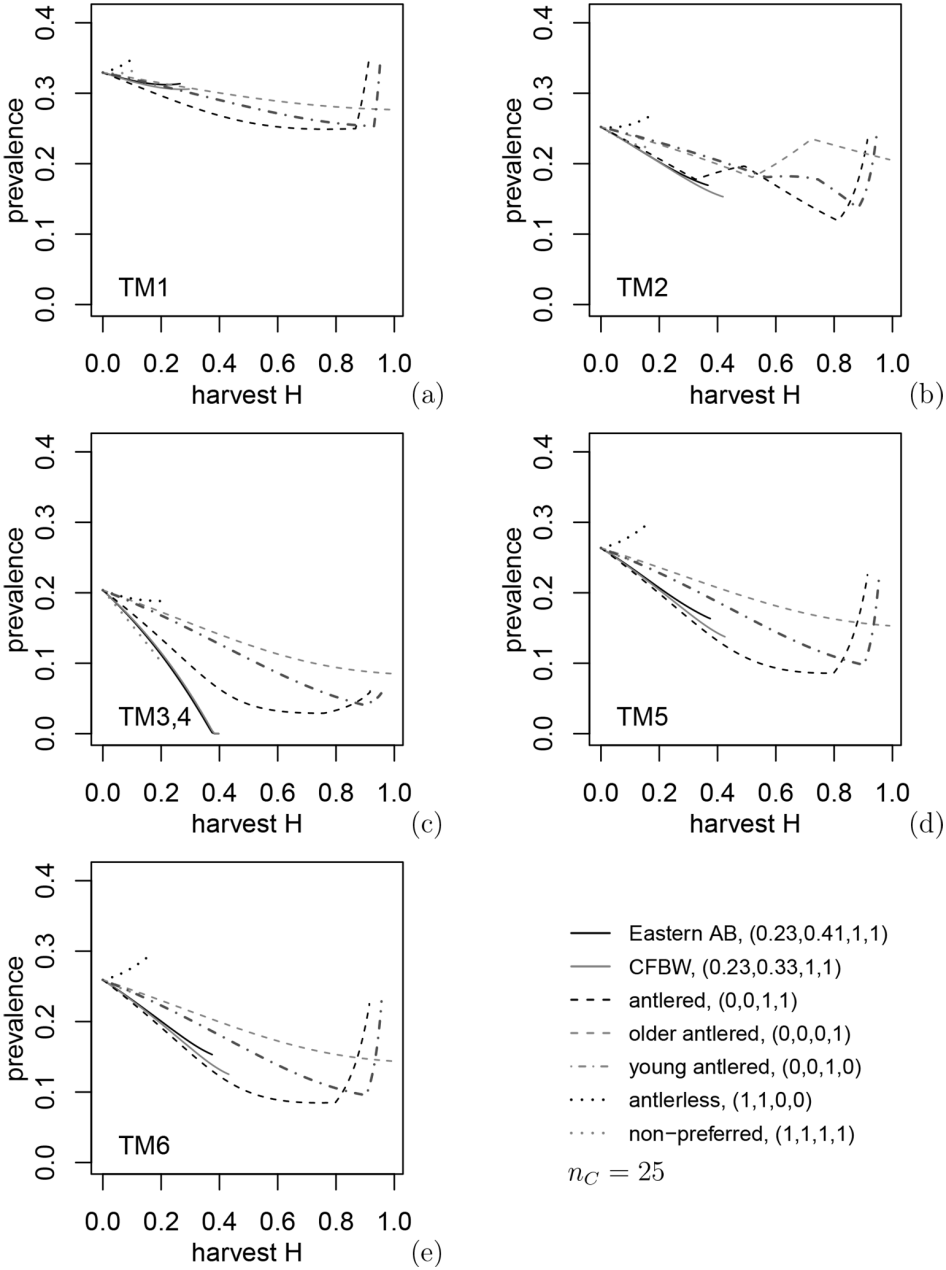
Dependence of efficiency of harvest control with fixed preferences on transmission mechanism TM*i* (bottom left corner) and preferences (*h*_*Pj*_, *h*_*Pf*_, *h*_*Pm*1_, *h*_*Pm*2_) shown by line styles. Non-monotonous behavior arises due to switching in the model: involvement of younger males in mating or too low buck:doe ratio and decline in birth rate. Density dependence is according to (A12), but (A14) with θ = 1 and 2 give indistinguishable plots.

In this section, we consider the effect of annual harvest proportion, *H*, from 0 to 0.91 (*h* = 10 year^−1^) or until the deer population collapses, and fixed preferences under the seven common harvest policies described above (Table 1). Disease prevalence under these policies differed, depending on disease transmission mechanism (Fig 1: panels a to f) and the harvest policy (Fig 1: different curves in each panel). Nonetheless, three common patterns emerge among the disease transmission mechanisms that are related to differences in disease transmission of juveniles. First, the best prevalence reduction is achieved for environmental transmission, TM3, 4 (panel c) under open general harvest in eastern Alberta and on CFBW. Under TM3, 4 juveniles are less frequently infected and produce less infection due to lower food consumption than adults, and deer density reduction increases the recruitment of new healthy adults [7]. Second, in other four TMs mechanisms based on direct transmission, juveniles are infected and spread the disease at a rate equal to that of adult females, the most efficient harvest regulation for reducing disease prevalence appears to be harvesting only antlered deer. Harvesting females just reduces the population viability. Third, harvesting only younger or older males gives less prevalence reduction compared to harvest of both antlered categories together, just because in the latter case the total number of removed individuals is greater (three dashed lines in Fig 1). Therefore, at the assumptions used, both younger and older males contribute significantly to the disease spread.

### 3.3 Optimal harvest policy: equal harvest for antlered classes, proportional harvest for antlerless classes

**Fig 2:**
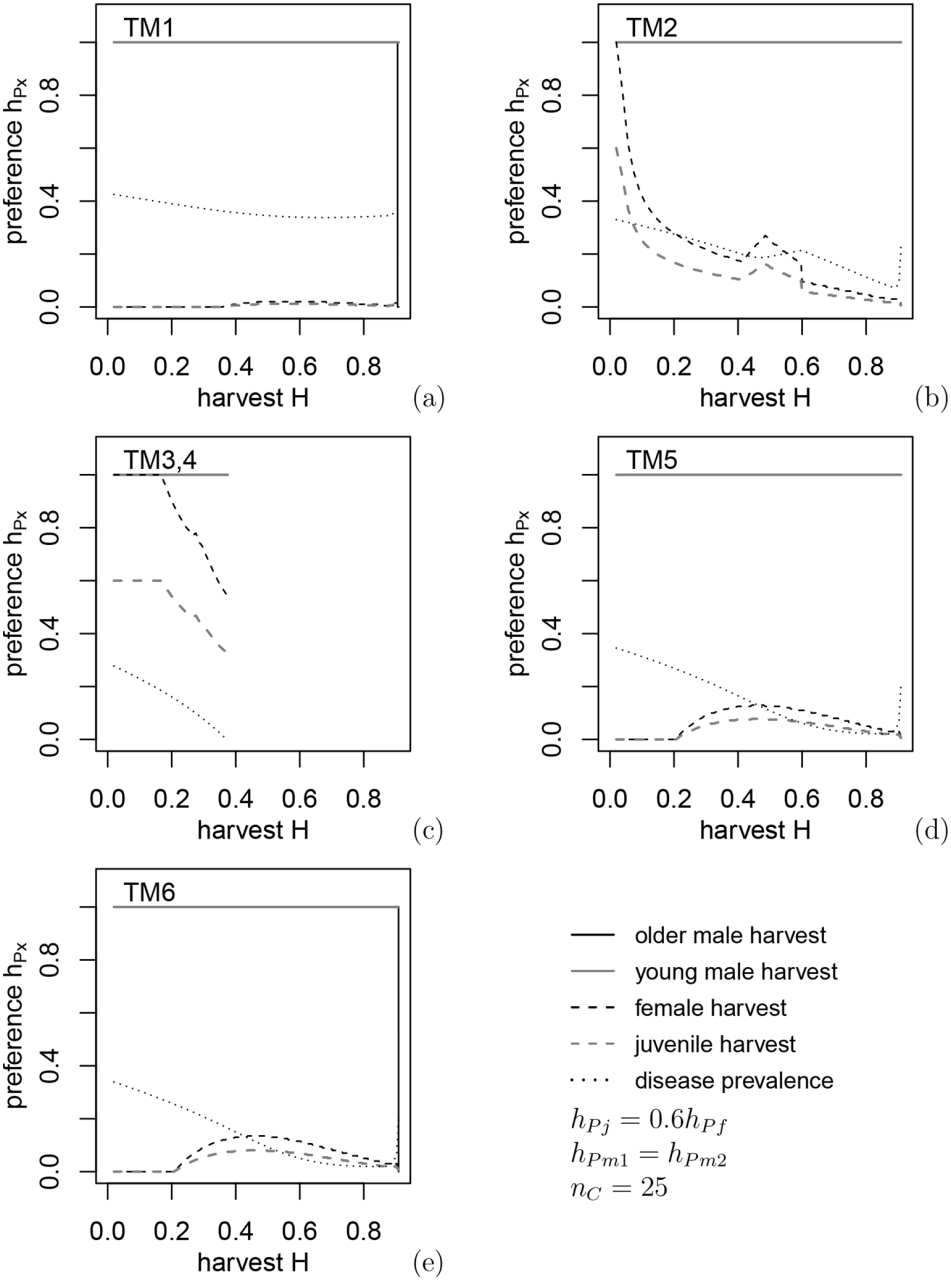
Optimal harvest preferences under constraints *h*_*Pj*_(*H*) = 0.6*h*_*Pf*_(*H*), *h*_*Pm*1_(*H*), *h*_*Pm*2_(*H*), which give the lowest disease prevalence at the given harvest intensity *H*, for the six different transmission mechanisms.

**Fig 3:**
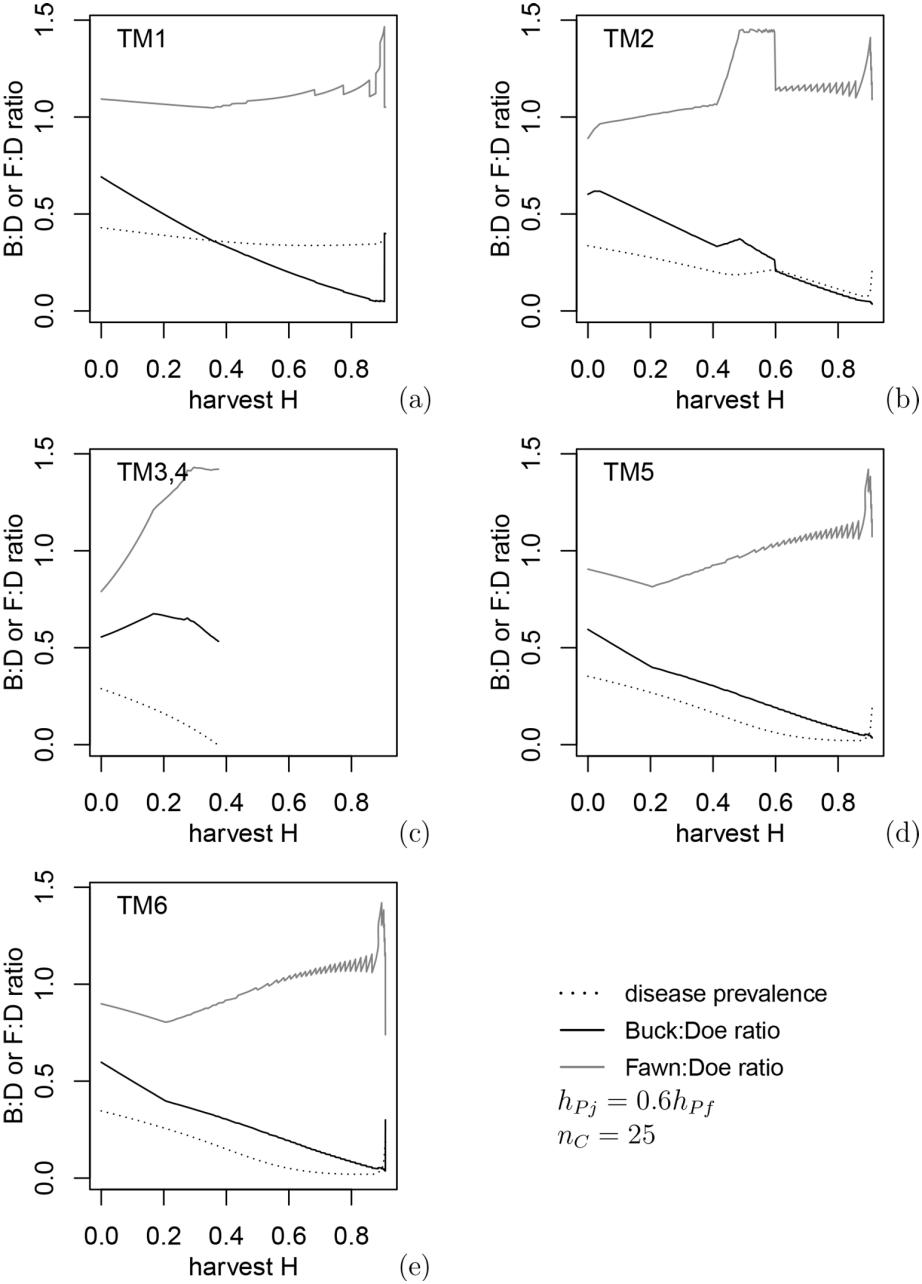
Buck:doe and fawn:doe ratios corresponding to constrained optimal harvest preferences in Fig 2.

In this section we find the best policy for reducing CWD prevalence for each harvest rate *H* that does not lead to the collapse of the deer population under the harvest policy of 1) equal harvest for all antlered deer regardless of age,*h*_*Pm*1_ = *h*_*Pm*2_ and 2) proportional harvest for females and juveniles *h*_*Pj*_ = 0.6*h*_*Pf*_, a relation similar to hunter preferences in open general seasons in eastern Alberta. The optimal harvest regimes (i.e., greatest reduction in CWD prevalence) for each transmission mechanism under varying harvest levels are shown in Fig 2 and the buck:doe and fawn:doe ratios corresponding to these control harvests in Fig 3, with both figures indicating the disease prevalence achieved as a result of harvest.

In contrast with Fig 1, CWD can be eradicated under environmental transmission (TM3,4) when either male food consumption is assumed to be higher than females and juveniles, or direct transmission to males is assumed to be higher due to being more susceptible (TM5) or males having higher contact rates (TM6). In all cases the eradication results in a lower buck:doe ratio due to buck removals and a higher fawn:doe ratio, which is related to disease dilution due to increased juvenile survival at low deer densities. For environmental transmission, the harvest level must be quite intensive to achieve low prevalence by evoking maximum density-dependent recruitment rates, which occurs only near population collapse due to lack of females. Environmental transmission to fawns is low, so the optimal policy is related to the maximum increase of fawn:doe ratio due to higher fawns survival at low densities (Fig 3). For transmission paths other than via the environment, the fawn:doe ratio under optimal harvest levels is almost twice as low compared to environmental transmission, and intensive harvest of females is not necessary. In contrast, CWD is not effectively eliminated under direct or indirect transmission only (TM1 and TM2: Fig 3); in fact, for TM2 (transmission to males from females during the rut) there is an interval of *H* values where harvest increases the prevalence. The reason for this is an increased fawn:doe ratio above 1 and growth of the disease prevalence in juveniles: the mechanism of disease dilution loses efficiency when disease transmission to juveniles is strong. In this case, classification of deer only as antlered and antlerless does not lead to the best harvest policy. Again, under all mechanisms, optimal control policies appear to be most efficient when control harvest targets males. However, approaching full eradication of the disease may require annual removal up to 80% of males.

**Fig 4:**
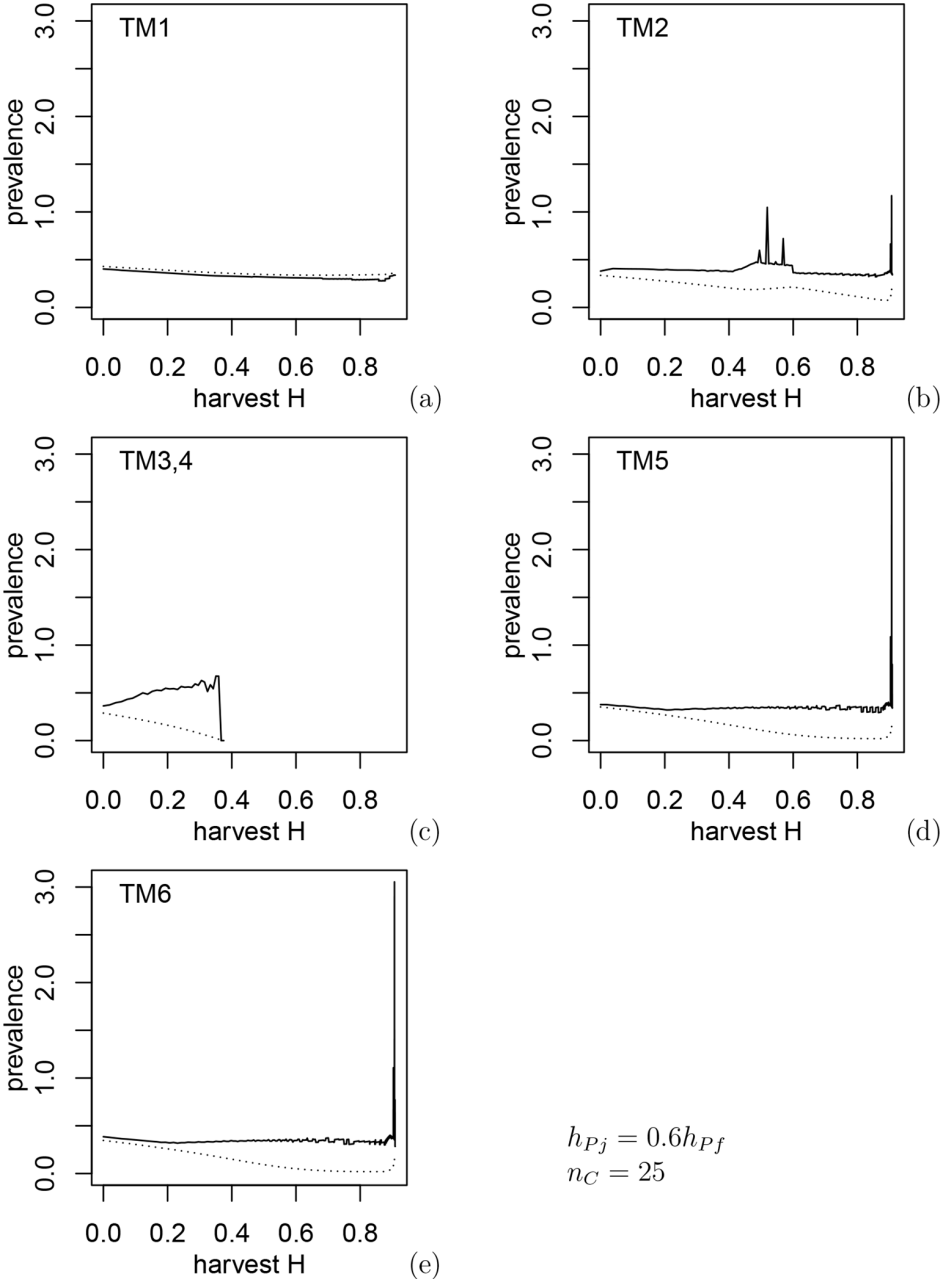
Ratio of juvenile to adult disease prevalence (solid line) and population disease prevalence (dotted line) corresponding to constrained optimal harvest preferences in Fig 2 and fawn:doe ratios in Fig 3. For TM3 and TM4 disease transmission to juveniles is less and optimal harvest regimes result in a higher proportion of juveniles in the population.

In Fig 4 where we compare juvenile and adult disease prevalence in the population for the six transmission mechanisms,results support the conclusion that one of the major differences in transmission mechanisms is related with disease transmission to juveniles. The ratio of juvenile to adult prevalence is the least for the case of environmental transmission (TM3,4) where juveniles consume less food and presumably are less exposed. However, it is noticeably higher for other TMs when juveniles have equal transmission rates to adults, since the disease dilution due to increased density-dependent juvenile survival is less efficient in this case or may not work at all.

### 3.3 Optimal harvest policy: independent harvest for three deer classes

**Fig 5:**
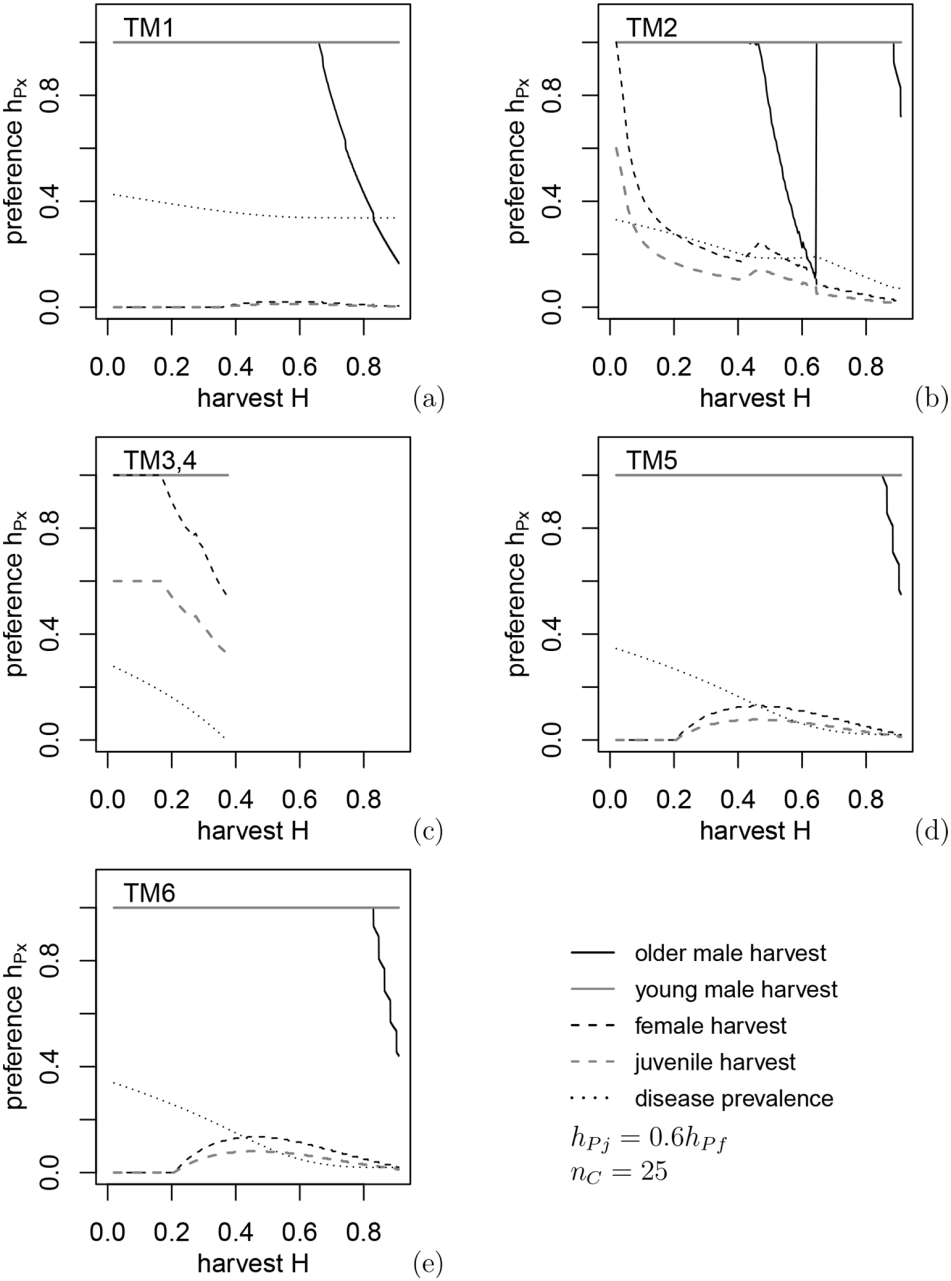
Optimization of harvest preferences. In contrast with Fig 2, *h*_*Pf*_(*H*), *h*_*Pm*1_(*H*), *h*_*Pm*2_(*H*) are selected independently. The control regimes are much more complicated compared to partial optimization in Fig 2, but typically this gives only a small decrease of disease prevalence. Partial or constrained optimization appears more practical.

**Fig 6:**
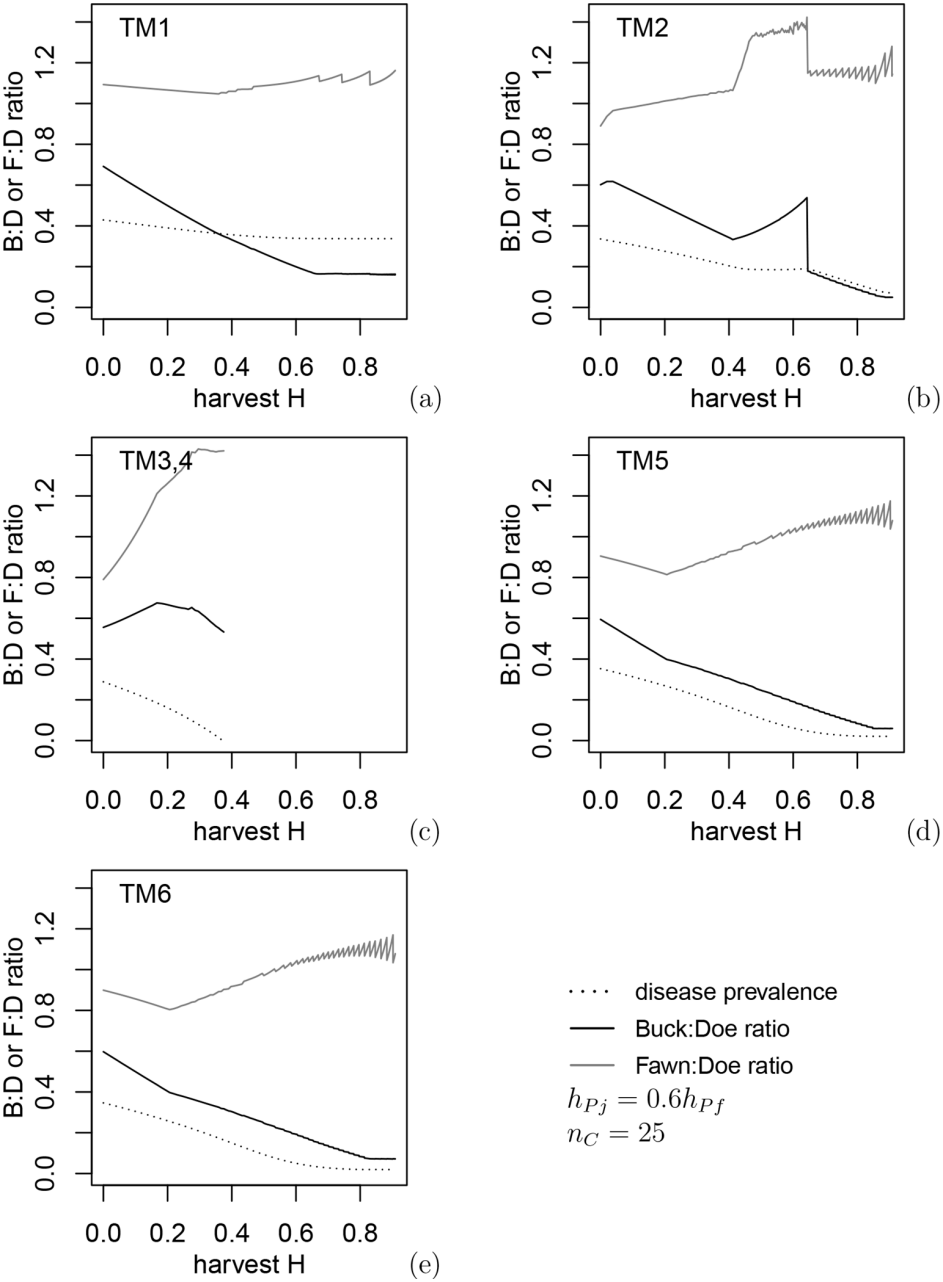
Buck:doe and fawn:doe ratios corresponding to the optimal harvest preferences in Fig 5. In spite of differences in control policy, the ratios are close to those in Fig 3, and disease eradication is achieved at the same harvest intensity as in Figs 2 and 3.

When harvest regulations for older adult males and younger adult males are devised independently, results show little difference in disease prevalence from these in the previous section but a substantial increase in complexity of the control (compare Figs 2 and 3 to Figs 5 and 6). Quality deer management, where harvest targets older males, provided a noticeable decrease in disease prevalence only for direct transmission including the rut (TM2) when *H* is between 0.3 and 0.6 (Fig 5b); however, the disease cannot be eradicated in this case. Results shown in Fig 1b indicate that in the case of TM2 there is a small interval of *H* values where harvesting young males reduces prevalence more than harvesting older or both classes of males.

Optimal harvest policies under both types of male harvest are often unstable with respect to small variations in *H*. Several policies often give very close values of prevalence, and a small change in *H* results in a different optimal policy. Nonetheless, comparison of Figs 3 and 6 show that buck:doe and fawn:doe ratios appear to be close whether the two age classes of males are harvested the same or differently. When male age classes are harvested independently, optimization produces a saw-like pattern in disease prevalence: a small change in harvest intensity gives a very different optimal policy, e.g. because a policy previously deemed optimal leads to deer extinction. Because of the complexity of these patterns, it is hard to formulate management recommendations consistent within a relatively wide interval of harvest intensities.

## 4. Discussion

### 4.1 Mechanisms of Disease Transmission and Harvest Management

We used a more detailed population model than that in [7] to evaluate nonselective (i.e., not focused on infected animals) harvest on control of CWD. We found that, for the fitted values of the transmission coefficients, harvest may reduce prevalence and sometimes even disease levels to approach eradication, even under FD transmission. However, we also found that a harvest policy that results in reduction in disease prevalence without population collapse (optimal policy) depends not only on the harvest intensity, but on the mechanisms of disease transmission. Because the major routes of CWD transmission in free-ranging populations remain unknown, this limits our ability to provide definitive recommendations for using harvest to control CWD. Nonetheless, several insights come from our modeling efforts.

First, the effect of harvesting antlerless deer on CWD prevalence depends on the transmission mechanism and the intensity of transmission to juveniles. Under environmental transmission, we assumed that food consumption was the major route of the disease spread through environmental contamination and that juveniles were less likely to be infected than adults because they eat less food that could be contaminated. Ingestion of infected soils, directly or indirectly, on plant material is most likely because current studies provide little evidence of the transport of CWD from roots to the stem and leaves of plants [22]. The low rate of infection of young is consistent with observed patterns of lower prevalence in fawns in general (e.g. [5]), but this may be related to exposure time. Any mechanism (environment, behavior, or susceptibility) that reduces exposure in juveniles is likely to contribute to a decline in prevalence when combined with a harvest that produces a density-dependent response favouring juvenile survival due to the dilution effect [7]. In fact, detailed information on the non-linearity in density-dependent recruitment, while generally lacking, is crucial if we are to fully understand how disease prevalence changes with density reductions under FD transmission.

Because stochasticity in juvenile survival can be high [13], we also used a mixed policy harvest strategy (see below) to test the sensitivity of the disease dilution mechanism to variation in juvenile survival. Stochasticity was introduced as variation in the amount of the available food (Section 2.3, see details in Appendix A and in Figure A9 in S1 File). It appears that stochasticity does not bring any new effects and its influence on the disease prevalence appears only at very intense harvest.

Second, although harvesting only males rarely reduced the population density sufficiently to promote a maximum increase of juvenile survival, our results indicate a control strategy that always includes relatively more male deer is most efficient because it removes the segment of the population with the highest proportion of infected individuals (e.g., [4,23,9]) due either to high food consumption, susceptibility, or other behavioural traits. Also simulations showed the importance of male-harvest strategies to maintain CWD-infected population in elk [24]. However, this generalization may depend upon the mechanisms we chose to include in this modeling exercise because they gave rise to a higher prevalence of CWD in males than females. None-the-less this is also is consistent with observations of prevalence in CWD-infected deer populations across a number of jurisdictions (e.g. [4,23,9]). If this assumption is correct, our results indicate that further fine-tuning harvest within the antlered segment of the population may not have significant effects. However, this outcome may be further contingent on the following model assumptions.

We assumed that the two antlered age classes did not differ in non-disease related mortality; if mortality were higher in older males, especially if a major route of transmission is sexual encounters during the rut (i.e., TM2 & TM4) and older males do most of the mating, the influence of their harvest on prevalence may be more pronounced than our results indicate. We also assumed all antlered males equally associated with other males; but if older males isolate themselves or gather in groups with only older males [25], differential harvest of antlered age classes might have more effect on prevalence than our models show. To assess this effect we a) increased mortality rates two-fold for the older male class and b) decreased contact rates two-fold for the older male class (See Appendix A and Figure A5 in S1 File). We found that harvesting more young males than adult males is only as efficient as harvesting all males only but not as efficient as older males (Figure A2 in S1 File). If older males have lower survival and remain more isolated from the population than young adult males, then harvest of young adult males could be a possible management alternative where the goal is simultaneously to retain some older males for quality deer management.

Past efforts in modeling CWD transmission based on empirical data indicate difficulty in distinguishing between frequency and density-dependent mechanism for its spread [3]. Our modeling shows that even if we determine CWD transmission as FD, details of the potential routes of FD could be critical to designing effective harvest management. The potential mechanisms of disease transmission we addressed were chosen in [6] to produce the ~2:1 male:female ratio of infection observed across a number of jurisdictions at equilibrium or initial conditions. The importance of mechanisms driving this sex-biased prevalence may not persist over time as prevalence builds up in the population.

### 4.2 Harvesting for CWD management

Key results of our modeling indicate an increase in harvest focused on males as the most reliable guideline at this time similar to what has been reported by Jennelle et al. [10]. Two alternative approaches exist to this guideline. First, because we do not know which of the 6 mechanisms dominates at the early stage of the disease spread, we can form a weighted policy that assigns prior weights to each of the optimal policies, e.g., 1/6, and sum up all policies with this weight. We formulated this approach based on our results and tested the mixed policy for all six TMs (Appendix A and Figure A8a in S1 File). The mixed policy is to harvest intensively both classes of males, while harvest preferences for juveniles and females decrease with *H*. Even when annual removal of males approached 80%, the proportion of annually removed females remained below 15% and for juveniles below 10% and did not cause the collapse of the deer population. Further, for 3 TMs it allows managers to reduce the disease, but only at very high values of annual harvest that may not be achievable without directed control programs.

In contrast, harvest strategy that results in partial selectivity of infected animals may be among the most efficient management tools. Selectivity effectively increases the disease-related mortality and reduces the number of secondary cases, see [7] for CWD-related analysis. For example, if disease prevalence is spatially structured, as inferred from genetic studies [26,27,28] or as apparent in hunter harvest data ([29,9,30] and M. Pybus, Alberta Fish and Wildlife, unpublished data), then spatially-based herd reduction programs adjacent to known hunter-killed CWD positive cases or taking out entire social groups (small scale spatial) may improve our chances of controlling the disease as has been the presumption of management for CWD in Illinois [13].

### 4.3 Final considerations

A key to assessing the effects of disease on wildlife populations is knowing transmission rates, which, in the case of CWD, are only now emerging [3]. The values of transmission coefficients for Alberta that we present here do not differ significantly from one reported by [3] despite the differences in our models. At present there is no evidence that an equilibrium state is achieved in wild populations; therefore, we cannot validate the modeling results using the equilibrium state. If the estimates of transmission coefficients are low, using harvest to reduce CWD in deer may require too intensive a removal to maintain a viable population and may become impossible to achieve once the disease is established widely. In this case an effective vaccine may be the only other control measure, despite the associated challenges [31,32,33], although there is some evidence that combining harvest with vaccination programs may be less efficient than vaccination alone because harvest removes vaccinated individuals as well [7].

Further, if dilution through increased recruitment is a major mechanism for decreasing CWD prevalence in deer, then in situations where populations are far below carrying capacity [6], either due to past disturbances or predation, herd reductions may not be effective at managing the disease if CWD transmission is primarily frequency dependent. Also, because adult females form a large portion of the population, very intensive doe harvests may be necessary to achieve a density-dependent increase in fawn survival. Encouraging hunters to harvest females is difficult and requires major incentives for success, such as “earn-a-buck” programs, because hunter preference for does typically is low [34].

Finally, we did not account for differential non CWD-related mortality in infected and uninfected deer in our models. Current data show infected deer have higher chances of being killed in vehicle collisions [35] and infected elk are more likely to be predated on by cougars [36] and infected deer may be more likely predated by wolves [37]. The same may be true for deer subject to human harvest; however, the direction of the bias is unclear because clinical behaviors may make deer more vulnerable (less responsive to danger) or less vulnerable (less active). We tested sensitivity of the optimal policies to this effect considering infected deer had 20% higher or lower chances to be harvested (see Figures A6 and A7 in Appendix A in S1 File). There were no qualitative differences in the harvest policies presented in Fig 4. Nevertheless, any positive selectivity of infected deer (more vulnerable to harvest) predictably allows managers to reduce the disease at lower harvest intensity [38]. This agrees with the modeling results under different assumptions presented in [37].

## 5. Conclusions

Efforts directed at modeling disease transmission to guide CWD control are now shifting away from the singular focus of determining whether the disease is frequency or density dependent recognizing it is likely both [39,40,10]. Our results indicate devising harvest strategies to manage for CWD control may be even more challenging than previously thought, requiring a basic understanding of dominant pathways of CWD transmission in a system. Nevertheless, because male deer seem to have the highest prevalence levels at least early in the epidemic, focusing harvest on male deer may be an effective approach and could be contributing to the relatively slow progression of the disease because most harvests already focus on male cervids [10]. We did not evaluate policies of selective harvest on infected individuals but first principles and preliminary field data indicate this this approach on selective harvest of infected individuals may be the best all-around approach [41]. In the face of the complexity and uncertainty about how the disease is transmitted, comparing the long-term outcomes of areas with different harvest policies may be the way to provide better guideline for areas where the disease has not yet gotten a foothold; however, this requires public support for devising alternative management policies and continuing surveillance programs.

## Supporting Information

S1 File Appendix A. Details of the model of deer population, deer harvest, and disease transmission. Appendix B. Mortality rate, compartments and the effective hazard function.
(PDF)
