# Supplementary material for: Chronic Wasting Disease: Transmission Mechanisms and the Possibility of Harvest Management
Source: PLoS One. 2016 Mar 10;11(3):e0151039. doi: 10.1371/journal.pone.0151039 (PMC4786122; doi:10.1371/journal.pone.0151039)
Supplement: S1 File — Appendix A. Details of the model of deer population, deer harvest, and disease transmission. Appendix B. Mortality rate, compartments and the effective hazard function. (PDF) [file pone.0151039.s001.pdf]

S1 File. Supporting Information

Appendix A. Details of the model of deer population, deer harvest, and disease transmission.

Equations of population dynamics

The model includes juvenile birth and maturation, natural mortality, harvest and disease transmission:

Rate of change	Birth or maturation	Natural mortality	Harvest	Disease transmission	
$\frac{dS_j}{dt} =$	$B\omega\left(\frac{D_m}{D_f}\right)\left[D_f - p_V I_f\right] - \tau^{-1}S_j$	$-\left(m_{0j} + Vm_{1j}\right)S_j$	$-h_j S_j$	$-\lambda_j S_j$	(A1)
$\frac{dS_f}{dt} =$	$0.5\tau^{-1}S_j$	$-m_{0f}S_f$	$-h_f S_f$	$-\lambda_f S_f$	(A2)
$\frac{dS_{m1}}{dt} =$	$0.5\tau^{-1}S_j - \tau_m^{-1}S_{m1}$	$-m_{0m}S_{m1}$	$-h_{m1}S_{m1}$	$-\lambda_{m1}S_{m1}$	(A3)
$\frac{dS_{m2}}{dt} =$	$\tau_m^{-1}S_{m1}$	$-m_{0m}S_{m2}$	$-h_{m2}S_{m2}$	$-\lambda_{m2}S_{m2}$	(A4)
$\frac{dI^{(1)}{}_j}{dt} =$	$p_V B\omega\left(\frac{D_m}{D_f}\right)I_f - \tau^{-1}I^{(1)}{}_j$	$-\left(m_{0j} + Vm_{1j} + \rho\right)I^{(1)}{}_j$	$-h_j I^{(1)}{}_j$	$+\lambda_j S_j$	(A5)
$\frac{dI^{(1)}{}_f}{dt} =$	$0.5\tau^{-1}I^{(1)}{}_j$	$-\left(m_{0f} + \rho\right)I^{(1)}{}_f$	$-h_f I^{(1)}{}_f$	$+\lambda_f S_f$	(A6)
$\frac{dI^{(1)}{}_{m1}}{dt} =$	$0.5\tau^{-1}I^{(1)}{}_j - \tau_m^{-1}I^{(1)}{}_{m1}$	$-\left(m_{0m} + \rho\right)I^{(1)}{}_{m1}$	$-h_{m1}I^{(1)}{}_{m1}$	$+\lambda_{m1}S_{m1}$	(A7)

$$\frac{dI^{(1)}{}_{m2}}{dt} = \tau_m^{-1} I^{(k)}{}_{m1} \quad -\left(m_{0m} + \rho\right) I^{(1)}{}_{m2} \quad - h_{m2} I^{(1)}{}_{m2} \quad + \lambda_{m2} S_{m2} \qquad \text{(A8)}$$

$$\frac{dI^{(k)}{}_j}{dt} = -\tau^{-1} I^{(k)}{}_j \quad -\left(m_{0j} + V m_{1j} + \rho\right) I^{(k)}{}_j \quad - h_j I^{(k)}{}_j \quad + \rho I^{(k-1)}{}_j \qquad \text{(A5')}$$

$$\frac{dI^{(k)}{}_f}{dt} = 0.5\tau^{-1} I^{(1k}{}_j \quad -\left(m_{0f} + \rho\right) I^{(k)}{}_f \quad - h_f I^{(k)}{}_f \quad + \rho I^{(k-1)}{}_f \qquad \text{(A6')}$$

$$\frac{dI^{(k)}{}_{m1}}{dt} = 0.5\tau^{-1} I^{(k)}{}_j - \tau_m^{-1} I^{(k)}{}_{m1} \quad -\left(m_{0m} + \rho\right) I^{(k)}{}_{m1} \quad - h_{m1} I^{(k)}{}_{m1} \quad + \rho I^{(k-1)}{}_{m1} \qquad \text{(A7')}$$

$$\frac{dI^{(k)}{}_{m2}}{dt} = \tau_m^{-1} I^{(k)}{}_{m1} \quad -\left(m_{0m} + \rho\right) I^{(k)}{}_{m2} \quad - h_{m2} I^{(k)}{}_{m2} \quad + \rho I^{(k-1)}{}_{m2} \qquad \text{(A8')}$$

$k = 2,...,n_C$, $\rho = n_C \mu$, where n_C is the number of disease-related mortality compartments, and μ^{-1} is the mean disease duration, so μ is the increase in mortality rate for a single-compartment mortality model. The total number of infected individuals in each deer category is obtained as a sum over all n_C compartments,

$I_x = I_x{}^{(1)} + I_x{}^{(2)} + ... + I_x{}^{(n_C)}$, $x = j, f, m1, m2$.

In this model juveniles are born by both healthy and infected females. Proportion of males:females in juveniles is assumed 1:1. Juveniles become adult females and young males at rate τ^{-1}. Young males become older males at rate τ_m^{-1}. All categories of deer are harvested, harvest rate may depend on deer category, but it does not depend on infection status, so harvest is nonselective. Susceptible deer become infected at the rate proportional to force of infection λ, which also depends on deer category.

The birth rate depends on buck:doe ratio D_m / D_f, $B\omega\left(D_m / D_f\right)$where the function $\omega(x)$ linearly grows for small *x* and then stabilizes at 1:

$$\omega(x) = \begin{cases} 1, & x \geq 0.05, \\ 20x, & 0 \leq x < 0.05. \end{cases} \quad \text{(A9)}$$

Part of the infected females may transmit infection to their fawns at birth or earlier, which is described by the probability of vertical transmission p_V.

Non-disease-related mortality and density-dependence

Per capita mortality rate for adults is assumed density independent [13] and equal to $m_{0S,f}$, $m_{0S,m}$, $m_{0I,f}$ and $m_{0I,m}$ respectively. Mortality for both male classes is assumed the same. For a single infected compartment, mortality rate of infected deer should increase by the inverse mean duration of the disease, which is about 2 years [1],

$$m_{0I,f} = m_{0f} + \mu, \quad m_{0I,m} = m_{0m} + \mu. \quad \text{(A10)}$$

For multiple infected compartments the increase is $\rho = n_C\mu$. The rate of passing through each compartment is greater, but the mean duration of the disease remains the same, see Fig. 1. In the literature there are two estimates of μ. The first one $\mu = 0.57\,\text{year}^{-1}$ has been done by Miller et al. [14] with fitting SI-type model to captive deer mortality data. The second one can be derived from the observation of Miller et al. (Miller MW, Swanson HM, Wolfe LL, Quartarone FG, Huwer SL, Southwick CH, Lukacs PM. Lions and prions and deer demise. PLoS one 2008; 3: e4019), that per year survival of infected free-ranging female mule deer is reduced by factor 0.53/0.82=0.64, which corresponds to $\mu = \ln(0.64)\,\text{year}^{-1} \approx 0.43\,\text{year}^{-1}$. Both estimates are close, and in the calculations below we use the first one.

We assume that the main way of natural deer population regulation is density-dependent juvenile mortality, which is usually related with predators and food limitation

during most critical season, which is winter for Alberta. We used two models of density dependence, and both of them are based on the ratio of required F_R and available F_A food for deer population. The required food is estimated with the help of daily food consumption rates $F_{S,x}$, $F_{I,x}$, $x = j, f, m$ for each deer category (see Table 1). Then

$$F_R(S_x, I_x) = \\ = C\left(F_{S,j}S_j + F_{S,f}S_f + F_{S,m}(S_{m1} + S_{m2}) + F_{I,j}I_j + F_{I,f}I_f + F_{I,m}(I_{m1} + I_{m2})\right), \quad \text{(A11)}$$

where C is a constant proportional to the duration of food-limiting period and the area occupied by the population. The available food is estimated from the same expression (A11) but for the densities of equilibrium population $S_{0j}, S_{0f}, ..., I_{0m2}$, which assumed to be known: $F_A = F_R(S_{0x}, I_{0x})$. Then in the ratio F_R / F_A the unknown constant C vanishes.

The first way of characterizing food limitation is through the value that we call starvation index V,

$$V = \max\left\{0,\ 1 - \frac{F_A}{F_R}\right\} \quad \text{(A12)}$$

[15]. If there is excessive food, i.e., the population is way below winter carrying capacity, then $F_A > F_R$ and V=0. If F_A is much less than F_R and starvation rates are high, V approaches 1. At a food-based equilibrium (at carrying capacity) V takes some value V_0 between 0 and 1, corresponding to partial food limitation. We assume that juvenile mortality is related with V as:

$$m_{vj} = m_{0vj} + Vm_{1vj}, \quad v = \{S, I\} \quad \text{(A13)}$$

where m_{0vj} corresponds to density-independent mortality and m_{1vj} to its density-dependent part. The model has been parameterized in [6] from deer survival data

presented in (White GC, Lubow BC. Fitting population models to multiple sources of observed data. Journal Wildlife Management 2002; 66:300-309). Data presented there represent mortality changes due to winter severity, but through food limitation as well. We assume that maximum per year fawn survival corresponds to *V*=0, the minimum one to *V*=1, and the equilibrium survival corresponds to V_0.

The second model of density dependent mortality is a generalization of expression given in [17]. It uses different expression for *V* in (A13),

$$V = \left(\frac{F_R}{F_A} \right)^{\theta}, \qquad \theta \geq 1. \tag{A14}$$

Below we used $\theta = 1$ and $\theta = 2$. This model has been parameterized by the same data: maximum survival corresponds to *V*=0 and equilibrium one to *V*=1. Note that the density independent part m_{0vj} for (A12) and (A14) should coincide, but the density dependent coefficients m_{1vj} are different.

Our modeling results do not show any influence of the chosen type of density-dependence in the model on disease prevalence and hence on the effect of harvest disease management. Choice of the density-dependence, however, may be critical for the study of the effect of harvest on deer density.

Density-dependence visualization (Fig. A1)

The goal is to visualize the behaviour of deer mortality and recruitment as density change. In general this is a complicated task, because the required food depends not only on density, but on proportions of deer of each categories. For this reason, we assume the

population healthy ($I_x = 0$) and the proportions $\pi_{Sx} = S_x / D$, $x = j, f, m1, m2$ fixed.

Then the required food is

$$F_R = \sum_x F_{Sx} S_x = D \sum_x F_{Sx} \pi_{Sx} ,$$

and similarly

$$F_A = D_0 \sum_x \left(F_{Sx} \pi_{Sx} \right),$$

where D_0 is the density of population used for model parameterizing. Therefore,

$$F_R / F_A = D / D_0 .$$

Substituting this for the expression for *V* and using (A13), we obtain Fig. A1a.

If in addition we assume that buck:doe ratio >0.05 and juveniles are not harvested, then at equilibrium

$$\frac{dS_j}{dt} = BS_f - \left(\tau^{-1} + m_{Sj} \right) S_j = 0, \qquad S_j = \frac{B}{\tau^{-1} + m_{Sj}} S_f ,$$

where m_{Sj} is density dependent juvenile mortality (A13), then per year recruitment

$$PER = S_j \tau^{-1} \times 1 \text{ year} = \frac{B\tau^{-1}}{\tau^{-1} + m_{Sj}} S_f \times 1 \text{ year} ,$$

and per female capita per year recruitment is

$$PFCYR = \frac{B\tau^{-1}}{\tau^{-1} + m_{Sj}} \times 1 \text{ year}$$

This gives us Fig. A1b.

Disease transmission

In [6] we develop the mathematical expressions for the force of infection terms for 7 transmission mechanisms and use four of them, which have been considered most plausible. They give the following force of infection terms for the 5 combination of age classes used in our harvest senarios:

$$\lambda_{m1,2} = \beta\left[w_S \frac{\psi_{mm} I_m}{S_m + I_m} + w_M \frac{\psi_{mm} I_m + \psi_{mf} I_f + \psi_{mj} I_j}{D} \right] + \lambda_{R1,2}, \tag{A15}$$

$$\lambda_f = \beta\left[w_S \frac{\psi_{ff} I_f + \psi_{fj} I_j}{S_f + I_f + S_j + I_j} + w_M \frac{\psi_{fm} I_m + \psi_{ff} I_f + \psi_{fj} I_j}{D} \right], \quad w_M + w_S = 2, \tag{A16}$$

$$\lambda_j = \beta\left[w_S \frac{\psi_{jf} I_f + \psi_{jj} I_j}{S_f + I_f + S_j + I_j} + w_M \frac{\psi_{jm} I_m + \psi_{jf} I_f + \psi_{jj} I_j}{D} \right]. \tag{A17}$$

$$S_m = S_{m1} + S_{m2}, \quad I_m = I_{m1} + I_{m2},$$

The two terms in square brackets in (A15)-(A17) correspond to disease transmission within sexually segregated groups (summer and early autumn) and within mixed groups (rut, winter and early spring). The weight factors w_S, w_M characterize proportional contribution of each type of seasonal deer behaviour to the total force of infection and are further described in [6]. The term $\lambda_{R1,2}$ in (A15) corresponds to disease transmission during mating or rut where:

$$\lambda_{R2} = \beta_R \frac{I_f}{S_{m2} + I_{m2}} L, \quad \lambda_{R1} = \beta_R \frac{I_f}{S_{m1} + I_{m1}} (1 - L), \quad L = \min\left\{1, \frac{10 D_{m2}}{D_f}\right\}. \tag{A18}$$

β and β_R are the corresponding transmission coefficients for within-group and rut transmission. For rut transmission, we assume that if there are enough older males, they alone mate with females, but if the density of the older males drops below one older male

per more than ten females, that is the ratio $D_{m2}/D_f < 0.1$, then younger males also take part in mating, which is described by L in (A18).

The matrix of coefficients ψ_{uv} characterizes relative intensity of transmission from infected deer of category v to susceptible deer of category u and changes with different assumptions about the principal route of disease transmission. We consider 5 combinations of transmission mechanisms that were identified in [6] as plausible. All of them, except for the null model, were capable of reproducing the observed difference in CWD prevalence between males and females found in many jurisdictions [4,5].

For transmission mechanisms in Table 1 we use the following assumptions:

TM1. (Null model) Direct transmission with equal intensity of contacts among all deer categories: $\psi_{uv} = 1$ and $\beta_R = 0$. Slightly higher prevalence in males may arise due to seasonality.

TM2. Direct transmission with equal intensity of contacts among males and females $\psi_{uv} = 1$ and significant mating transmission with $\beta_R = \beta$. Higher prevalence in males arises due to combination of mating transmission and seasonality.

TM3. Environmental transmission proportional to food consumption of both infected and healthy individuals. In this case $\beta_R = 0$ and

$$\psi_{uv} = \frac{F_{I,v} F_{S,u}}{\max_{x,y}\{F_{I,x} F_{S,y}\}}. \qquad \text{(A19)}$$

Higher prevalence in males arises due to higher food consumption of males than females.

TM4. Environmental and rut transmission. ψ_{uv} is defined by (A19) and $\beta_R = \beta$.

TM5. Direct transmission with equal intensity of contacts, but males are more susceptible to infection than females or juveniles. We introduce the ratio of male to female susceptibility $Y_m > 1$. Then $\psi_{mv} = Y_m$, $\psi_{fv} = \psi_{jv} = 1$.

TM6. Direct transmission with higher intensity of male-male contacts. All $\psi_{uv} = 1$ except for $\psi_{mm} = Z_m > 1$. Mathematically this is close to the case TM3, where ψ_{mm} is greater than all other entries, but interpretation is different.

Hunter harvest: annual removal

Harvest rates $h_x, x = j, f, m1, m2$, reflect the following. The deer population is surveyed once a year in January, when the hunting season is over, but before males lose their antlers. We interpret the deer densities D_x given by the model as the estimated population density at the time of the winter survey. At the state of equilibrium, when all deer densities do not change from year to year, the annual harvest rate for the deer of category *x* is $D_x h_x \Delta t$ deer/km^2 ($\Delta t = 1\,\text{year}$). Therefore, the pre-harvested deer density is $D_x + D_x h_x \Delta t$, and hence the proportion of the pre-hunted deer of the category *x* removed by harvest is

$$H_x = \frac{D_x h_x \Delta t}{D_x + D_x h_x \Delta t} = \frac{h_x \Delta t}{1 + h_x \Delta t}\,. \qquad \text{(A20)}$$

At a low harvest rate when $h_x \Delta t << 1$, $H_x \approx h_x \Delta t$, and hence h_x is approximately the proportion of category *x* removed per year. In other cases harvest intensity h_x and proportion of individuals removed annually H_x are related as

$$h_x = \frac{H_x}{1 - H_x} \text{ year}^{-1} . \quad \text{(A21)}$$

Disease-related mortality: multiple compartments

The reason for introducing multiple compartments for infected individuals is explained in Section 2.5: to reduce variability in infected deer survival times. This is illustrated in Fig. A2. Mathematical explanations are given in Appendix B. Here we discuss the effects that we observed comparing the results for different numbers of compartments n_C .

We fitted transmission coefficients for models with $n_C = 1, 5, 25$ to the same Alberta data for the initial stage of the disease. The results are shown in Table A3. An interesting effect is observed: the greater is n_C , the smaller is the fitted transmission coefficient. This may be the result of slower decrease in infected deer density in the first 1-1.5 years of the disease development. The total number of the secondary infections may be the same, but they are created in a shorter period. Therefore, the same initial disease dynamics can be reproduced with less intensive transmission.

The difference in disease transmission coefficients may create significant difference at the developed stage of the disease. For $n_C = 1$ and transmission model TM1 the population appeared to be at the edge of extinction with the adult prevalence close to 57%. At the same time, at $n_C = 25$ the predicted adult prevalence is about 40%, and the population remains viable. At present, there are no experimental data to make the correct choice of n_C , and we have chosen $n_C = 25$ due to closer resemblance to the reported disease duration in the literature. Note that for $n_C > 10$ there is very little dependence of

the results on n_C. Analogs of Fig. 2 for $n_C = 1$ and $n_C = 5$ are presented in Figs. A3 and A4 respectively. Comparison with Fig. 2 shows that the disease prevalence at the same harvest level is higher at smaller n_C. At the same time, optimal harvest policy remains practically the same.

Experiments with Healthier older male class: influence on harvest policies

In some publications on CWD, see [4,5], there are data that can be interpreted such that male disease prevalence grows with deer age reaching its maximum around 5 years old, and then decreasing. If disease transmission rates and mortality of adult male deer do not depend on age, as in our model, this can not happen: older males have higher disease prevalence just because of longer exposure to the infection. However, the older male class may have less contact rate because of less participation in bachelor groups, or higher mortality e.g. because of poorer condition in the beginning of the winter due to starvation and exhaustion during rut. In either of cases their prevalence may be less or not higher than that of young males, and there appears one more reasonable harvest strategy: harvest of young males only. We did a series of model calculations for the original model and the cases of twice less contact rate and twice greater mortality rate for the older male class. We searched for optimal harvest strategy with three independent harvest rates: antlerless, young males and older males. The optimal policies were less stable than in Figs. 2-3, and in most stable cases were close to that for the original model. However, the results of comparing the seven harvest policies were more interesting. For the transmission mechanisms TM3 (environmental) and TM5 (higher male susceptibility)

they are shown in Fig. A5. One can see that in case of healthier old male class (panels c-f) the results of harvesting young males only give slightly higher than harvest all males, but close to them. If the older male class is indeed healthier, then harvesting only young males could be an alternative management policy provided there is a goal to preserve older males.

Another interesting effect related with the hypothesis of healthier older male class is that fitting the model with twice less contact rate to data gives much higher value of rut transmission coefficient for the mechanism TM2 ($\beta_R = 0.72$) and TM4: ($\beta_R = 0.39$). The model compensates slower transmission to older males from other males by more intensive transmission from females.

Table A1. Notation for model variables and parameters.

Variable or subscript	Symbol	Units
Deer age and sex classes: males, females, juveniles	f, $m1$, $m2$, j	
Deer population density	D	deer/km^2
Density at disease-free equilibrium	D_0	deer/km^2
Density of susceptible males, females, juveniles	S_{m1}, S_{m2}, S_f, S_j	deer/km^2
Density of infected males, females, juveniles	I_{m1}, I_{m2}, I_f, I_j	deer/km^2
Fertility rate of healthy females	B	year^{-1}
Probability of vertical transmission	p_V	
Fertility rate of infected females for bringing healthy and infected fawns	$B_{IS}=(1-p_V)B$, $B_{II}=p_V B$	year^{-1}
Fawns maturation rate (inverse of juvenile stage duration τ,)	τ^{-1}	year^{-1}
Male aging rate (inverse of young adult stage duration τ_m,)	τ_m^{-1}	year^{-1}

Density-independent portion of per capita mortality rate	m_{0m}, m_{0f}, m_{0j},	year^{-1}
Starvation index	V	
Density-dependent portion of mortality rate of juveniles only	Vm_{0j}	year^{-1}
Per capita hunting rate (equal for S and I)	h_{m1}, h_{m2}, h_f, h_j	year^{-1}
Overall harvest rate	$h = \max\{h_{m1}, h_{m2}, h_f, h_j\}$	year^{-1}
Hunter's preferences	$h_{Pm1}, h_{Pm2}, h_{Pf}, h_{Pj}$, $h_x = h \times h_{Px},\ x = m1, m2, f, j$	
Per capita food consumption	$F_{S,m}$, $F_{S,f}$, $F_{S,j}$, $F_{I,m}$, $F_{I,f}$, $F_{I,j}$,	kg/day
Total force of infection (per susceptible capita disease transmission rate)	λ_m, λ_f, λ_j	year^{-1}
Disease transmission coefficient	β	year^{-1} or km^2/year
Seasonal weights	w_M, w_S	

Table A2. Parameter values used in modeling deer population dynamics

Parameter	Mule Deer	Comment
Birth rate for healthy females *B* (fawns per adult female)	1.65	Alberta Fish & Wildlife, unpublished data, Edmonton, AB
Maturation time τ	1.5 years	
Food consumption by healthy adult male, female, fawn air dry food kg/day $F_{S,m}$, $F_{S,f}$, $F_{S,j}$. (estimates in Appendix)	1.40, 1.09, 1.03	Potapov et al. (2013)
Food consumption by infected adult male, kg/day $F_{I,m}$	$=0.7F_{S,m}$	
Food consumption by infected adult female, kg/day $F_{I,f}$	$=0.7F_{S,f}$	
Food consumption by infected fawn, kg/day $F_{I,j}$	$=F_{S,j}$	
Equilibrium deer density for WMU 234, deer per km^2, D_0	1.58	Alberta Fish & Wildlife, unpublished data, Edmonton, AB<br>Habib et al. (2010)
Equilibrium proportions of healthy population S_{0m}/D_0, S_{0f}/D_0, S_{0j}/D_0	0.44, 0.18, 0.38	Potapov et al. (2013)
Adult female mortality m_{0f}	0.15	Potapov et al. (2013)

		Potapov et al. (2013)
Adult male mortality m_{0m}	0.29	Potapov et al. (2013)
Juvenile mortality $m_{0j}+Vm_{1j}$	0.30+12.3V	Potapov et al. (2013)
Mortality coefficient for infected adult males, years^{-1}, $m_{0I,m}$	m_{0m}+0.57	Miller et al. (2006)
Mortality coefficient for infected adult females, years^{-1}, $m_{0I,f}$	m_{0f}+0.57	Miller et al. (2006)
Mortality coefficient for infected fawns, years^{-1}, $m_{0I,j}$	=m_{0j}+0.57	(***)
Density-dependent mortality coefficient for infected adult males, years^{-1}, $m_{1I,m}$	0	Miller et al. (2006) (***)
Density-dependent mortality coefficient for infected adult females, years^{-1}, $m_{1I,f}$	0	Miller et al. (2006) (***)
Density-dependent mortality coefficient for infected fawns, years^{-1}, $m_{1I,j}$	=m_{1xj}	(***)
Hunters' preference for males, h_{Pm}, h_{Pf}, h_{Pj}	1.00, 0.33, 0.23	Potapov et al. (2013)

(***) No known evidence on difference with healthy deer

Table A3. Transmission mechanisms used in calculations, fitted transmission coefficients(β and one of w_S, β_R, Y_m, Z_m), and population characteristics at the developed stage of the disease (adult prevalence π_a and male/female prevalence ratio r_{mf}) for the number of disease-related mortality compartments $n_c = 1, 5, 25$.

Transmission mechanism	Direct/Indirect transmission and seasonality	Rut transmission	$n_c = 1$	$n_c = 5$	$n_c = 25$
**TM1**	Equal transmission between all deer classes ($\psi_{uv} = 1$); w_S fitted.	No	$\beta = 0.77$<br>$w_S = 1.71$<br>$\pi_a = 0.58$<br>$r_{mf} = 1.05$	$\beta = 0.63$<br>$w_S = 1.67$<br>$\pi_a = 0.44$<br>$r_{mf} = 1.14$	$\beta = 0.60$<br>$w_S = 1.68$<br>$\pi_a = 0.41$<br>$r_{mf} = 1.18$
**TM2**	Equal transmission between all deer classes ($\psi_{uv} = 1$); $w_S = 1$	Yes	$\beta = 0.63$<br>$\beta_R = 0.60$<br>$\pi_a = 0.42$<br>$r_{mf} = 1.27$	$\beta = 0.52$<br>$\beta_R = 0.49$<br>$\pi_a = 0.33$<br>$r_{mf} = 1.47$	$\beta = 0.49$<br>$\beta_R = 0.48$<br>$\pi_a = 0.30$<br>$r_{mf} = 1.56$
**TM3**	Food-mediated transmission (high to males, medium to females, low to juveniles); w_S fitted.	No	$\beta = 1.05$<br>$w_S = 1.03$<br>$\pi_a = 0.29$<br>$r_{mf} = 1.65$	$\beta = 0.86$<br>$w_S = 1.02$<br>$\pi_a = 0.24$<br>$r_{mf} = 1.81$	$\beta = 0.82$<br>$w_S = 1.02$<br>$\pi_a = 0.23$<br>$r_{mf} = 1.86$
**TM4**	Food-mediated transmission (high to males, medium to females, low to juveniles); $w_S = 1$	Yes	$\beta = 1.05$<br>$\beta_R = 0.03$<br>$\pi_a = 0.29$<br>$r_{mf} = 1.65$	$\beta = 0.85$<br>$\beta_R = 0.03$<br>$\pi_a = 0.24$<br>$r_{mf} = 1.82$	$\beta = 0.80$<br>$\beta_R = 0.04$<br>$\pi_a = 0.23$<br>$r_{mf} = 1.90$

**TM5**	Equal transmission between all deer classes, but male susceptibility is higher ($Y_m > 1$); $w_S = 1$	No	$\beta = 0.63$<br>$Y_m = 1.57$<br>$\pi_a = 0.44$<br>$r_{mf} = 1.34$	$\beta = 0.52$<br>$Y_m = 1.55$<br>$\pi_a = 0.34$<br>$r_{mf} = 1.52$	$\beta = 0.49$<br>$Y_m = 1.56$<br>$\pi_a = 0.32$<br>$r_{mf} = 1.58$
**TM6**	Increased male-to-male transmission ($Z_m > 1$), equal transmission between other deer classes; $w_S = 1$.	No	$\beta = 0.63$<br>$Z_m = 1.68$<br>$\pi_a = 0.43$<br>$r_{mf} = 1.31$	$\beta = 0.52$<br>$Z_m = 1.65$<br>$\pi_a = 0.33$<br>$r_{mf} = 1.49$	$\beta = 0.49$<br>$Z_m = 1.66$<br>$\pi_a = 0.31$<br>$r_{mf} = 1.55$

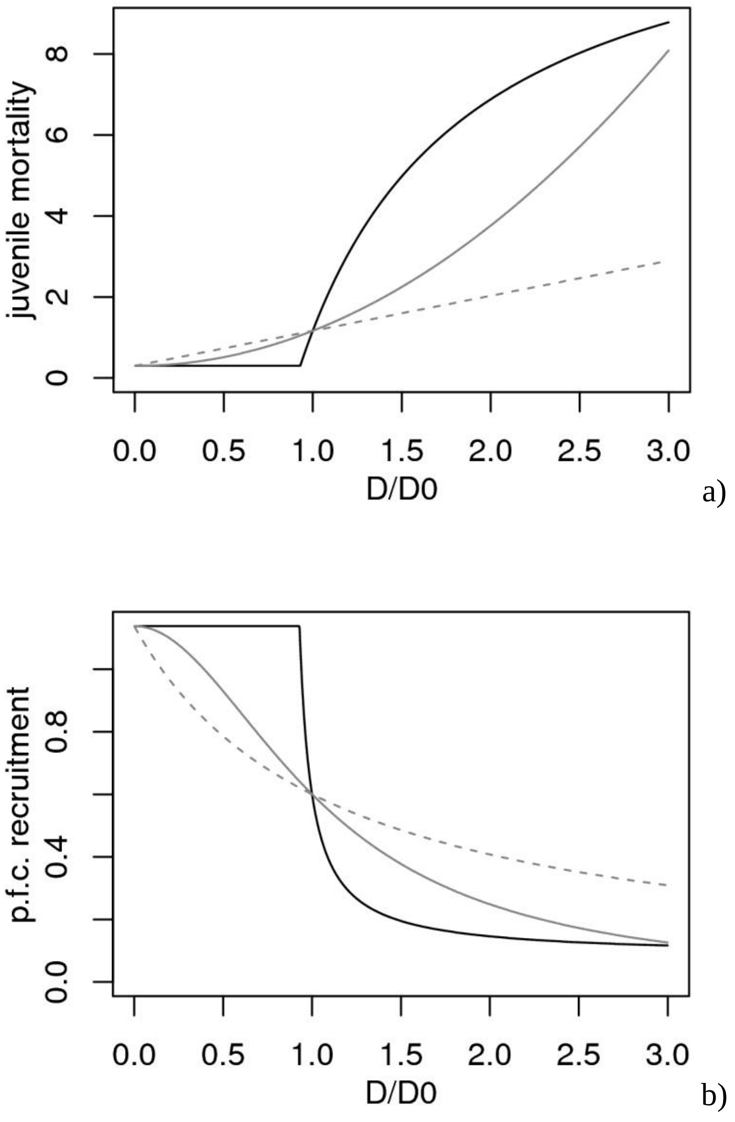

Fig. A1. Illustrations of juvenile mortality rate and per female capita recruitment rate vs deer density D/D_0 for three density-dependent juvenile mortality models. The curves are drawn under some simplifications, see text. Black solid line shows starvation model, grey lines correspond to logistic-like per capita mortality with linear (dashed, $\theta = 1$) and quadratic (solid, $\theta = 2$) density dependence.

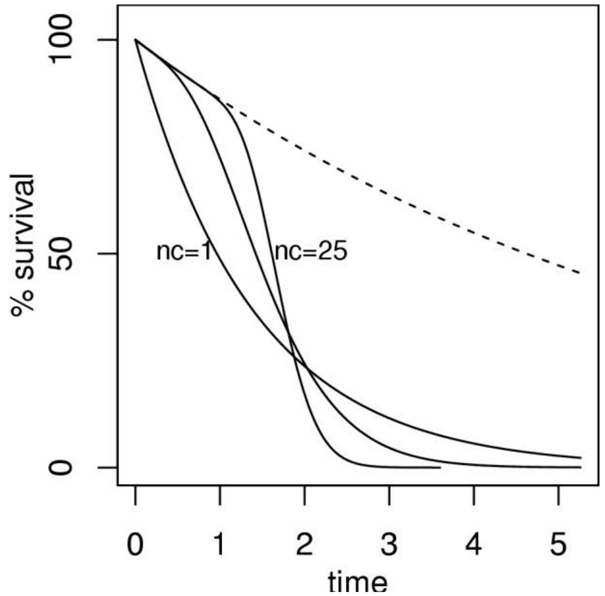

Fig. A2. An example showing the difference between three disease-related mortality models: % of adult female population surviving by certain time. Dashed line: healthy adult females. Solid lines: infected adult females, model with $n_c = 1$, 5 and 25 compartments. All three solid lines correspond to the same mean disease duration $\mu^{-1} = 1.75$ years.

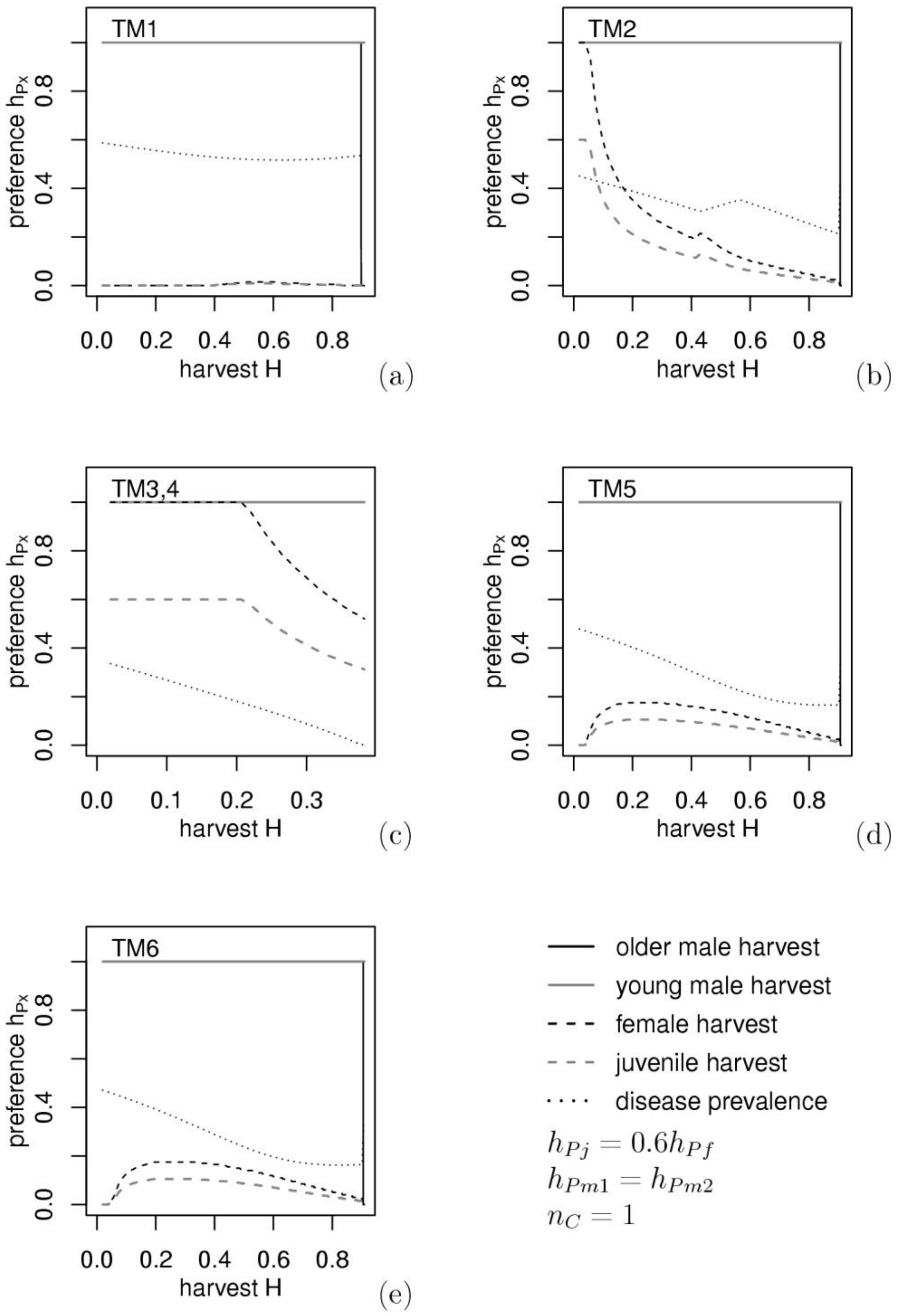

Fig. A3. Optimal harvest for 1-compartment disease-related mortality model.

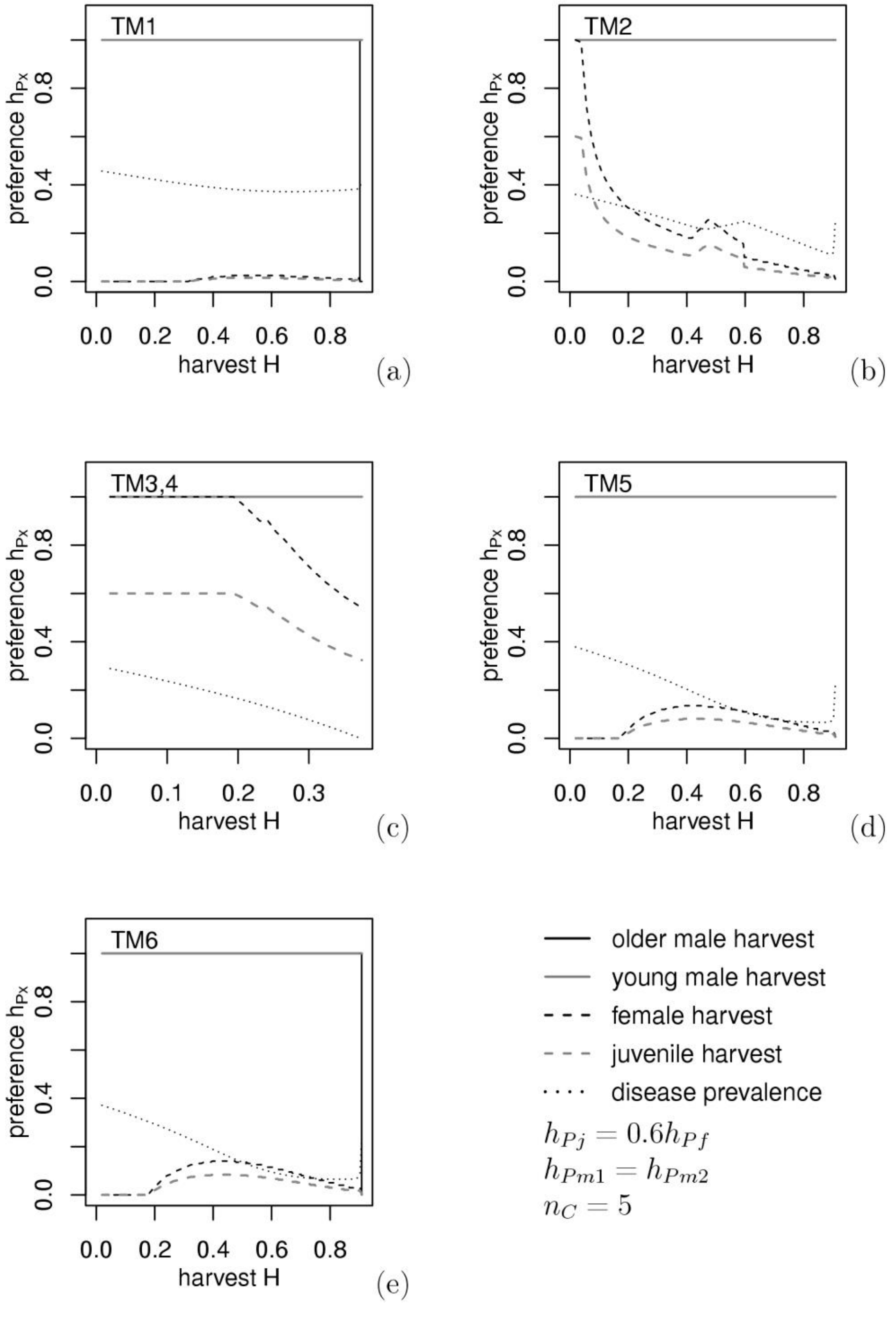

Fig. A4. Optimal harvest for 5-compartment disease-related mortality model.

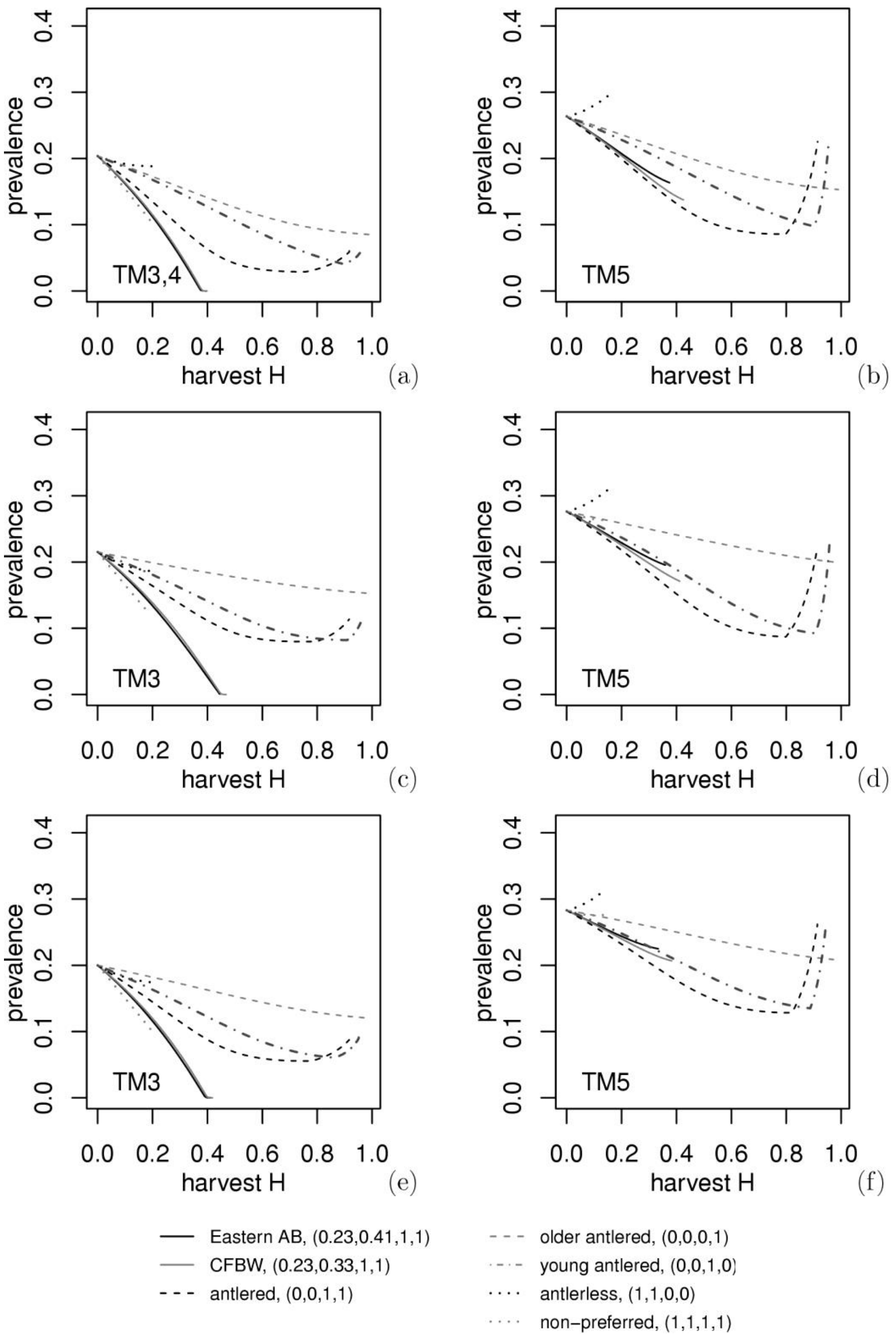

Fig. A5. Effect of only young male harvest for TM3 and TM5 in case of original model (a,b) (Fig. 3), twice less contacts for older males (c,d) and twice greater mortality rate for older males (e,f). In panels c-f harvest of young adults is closer by the effect to harvest of all adults.

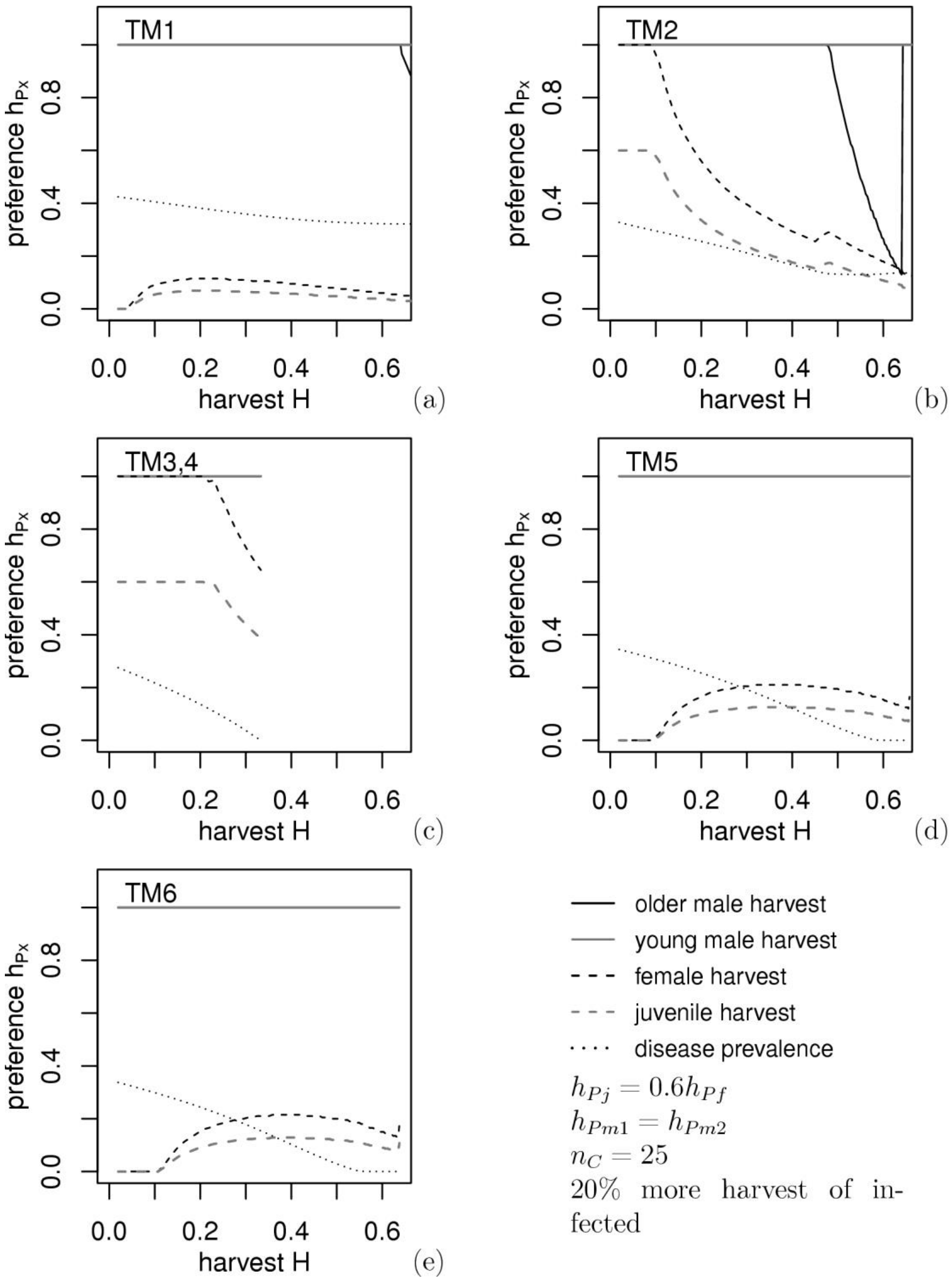

Fig. A6. Optimal policy if infected individuals are always harvested at the rate 20% greater than healthy ones (positive selectivity of infected).

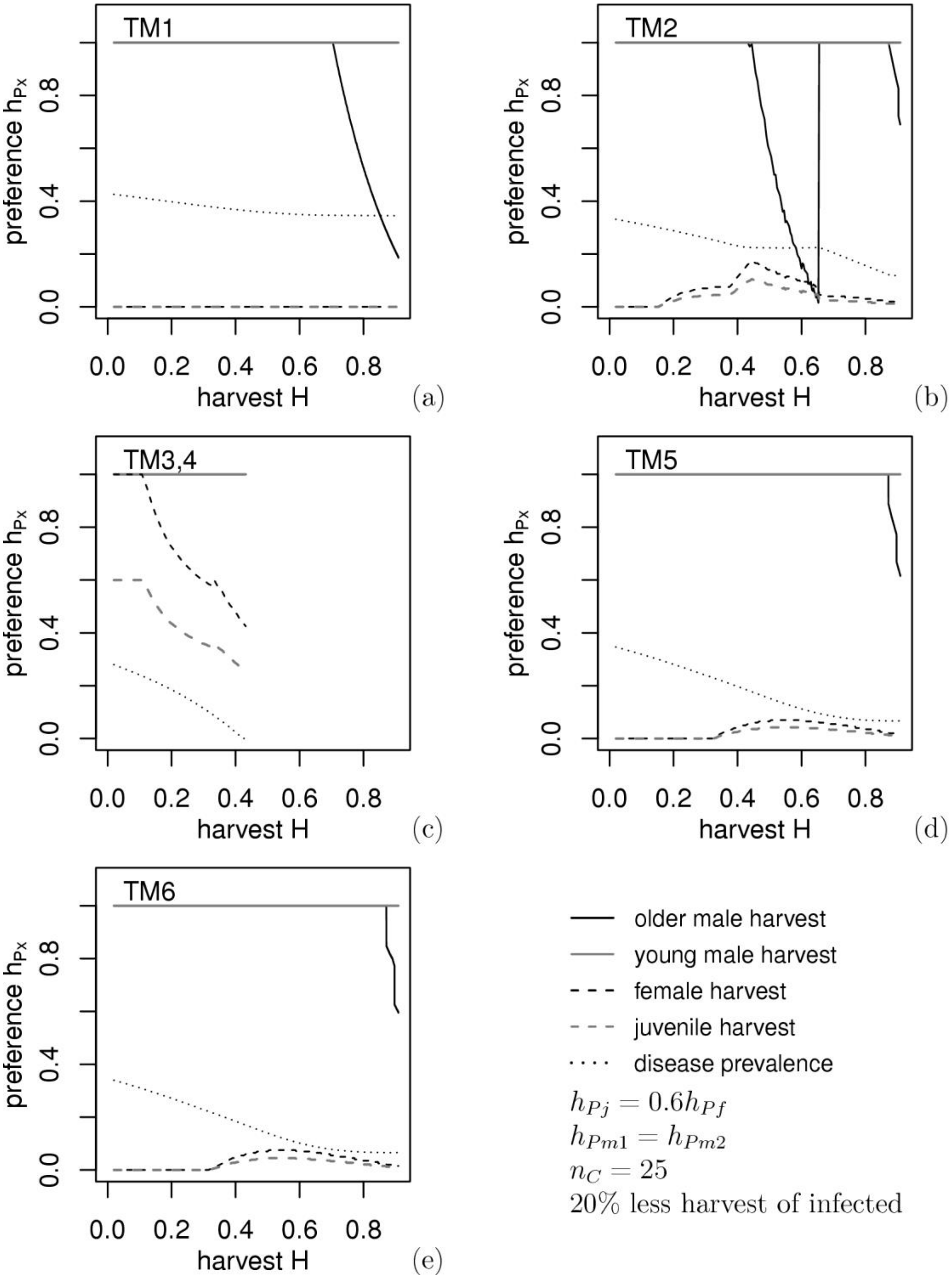

Fig. A7. Optimal policy if infected individuals are harvested at the rate 20% less than healthy ones (negative selectivity of infected).

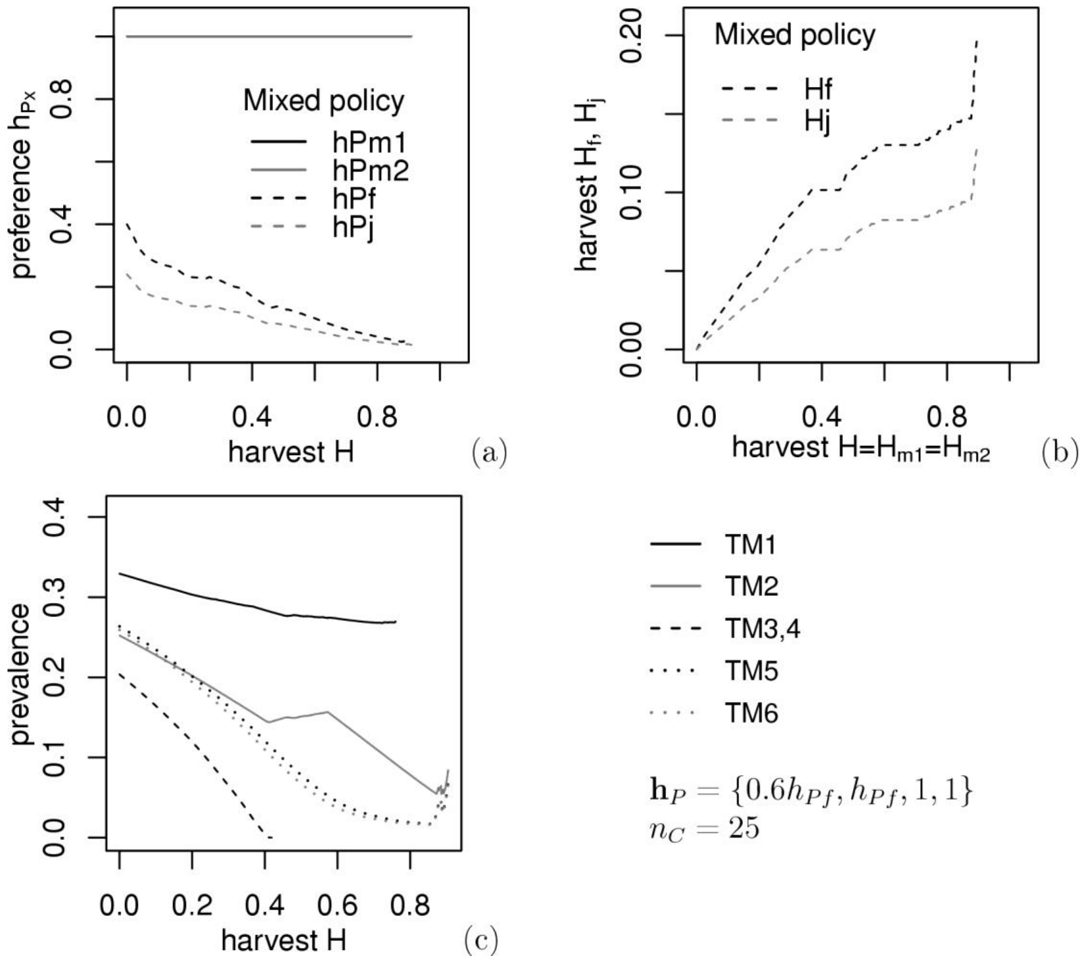

Fig. A8. a) Mixed policy (average of optimal ones for all six TMs shown in Fig. 4). b) Proportions of removed females and juveniles vs proportion of removed males for this policy. c) The disease prevalence under the mixed policy for each of TMs: for TM3, TM5 and TM6 the disease can be eradicated, but this requires greater harvest effort.

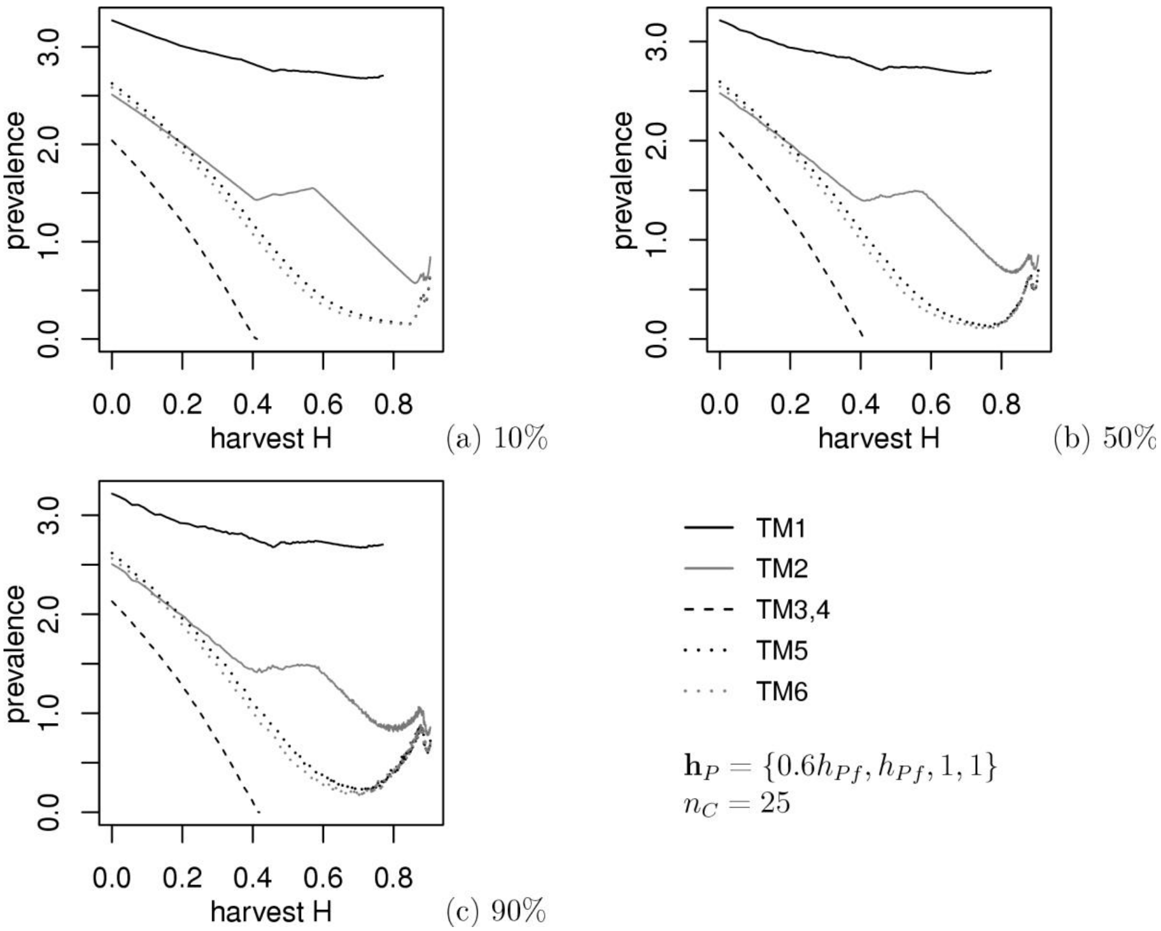

Fig. A9. a) Mixed policy shown in Fig. A5 with added stochasticity in available food F_A in (A12) and hence in juvenile mortality (A13). For 10% F_A variability (panel a), the results are very close to Fig. A5c. Stronger variability makes control less efficient. Since steady state does not exist with randomness added, shown is the disease prevalence averaged over 1000 years.

Appendix B. Mortality rate, compartments and the effective hazard function

This is an attempt to overcome the problem of nonconstant hazard function. Let us assume that on average individuals live about T=2 years since getting infected, then die. We shall split this interval in a number of compartments n, each of the compartments of the mean duration T/n. This corresponds to the rate of compartment leaving $\rho = n/T$. At t=0 let there be N_0 infected individuals in the first compartment.

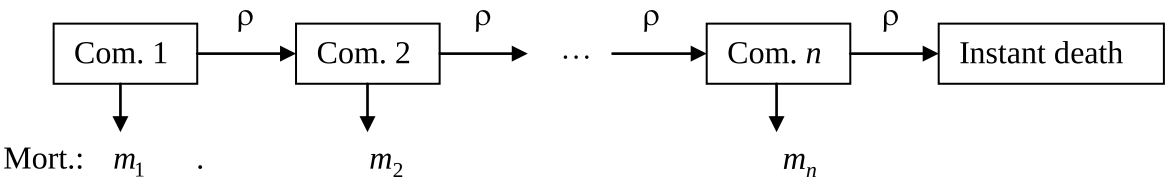

We consider only the destiny of these N_0 infected individuals, no reproduction. Within each compartment there may be additional death rate, not related with the disease, e.g. harvest or predation, for i-th compartment we denote it by m_i. The current population size within each of the compartments we denote by x_i. At t=0 $x_1(0) = N_0$, $x_i(0) = 0,\ i = 2,...,n$ The total population size is $N = x_1 + x_2 + ... + x_n$. This system is described by the equations:

$$
\begin{aligned}
& dx_1/dt = -(\rho + m_1)x_1, \\
& dx_2/dt = \rho x_1 - (\rho + m_2)x_2, \\
& ... \\
& dx_n/dt = \rho x_{n-1} - (\rho + m_n)x_n.
\end{aligned}
$$

This system can be solved analytically, however analytical solution in most cases becomes very bulky. In the simplest case $m_1 = m_2 = ... = m_n = m$ the solution has the simplest form,

$$x_k(t) = N_0 \frac{(\rho t)^{k-1}}{(k-1)!} \exp(-(\rho + m)t),$$

which is the Gamma-distribution.

We characterize the dynamics of the total population $N(t) = x_1(t) + x_2(t) + ... + x_n(t)$ by the survival rate $N(t)/N_0 \times 100\%$ and by the effective hazard function. By definition, hazard function is the probability of individuals to die between *t* and *t*+*dt* divided by *dt* provided they survived till the moment *t*. In other words, this is just the absolute instant relative growth rate $Hz(t) = |dN/dt|/N$.

We consider the cases $n = 1$, 2, 3, 4, 6, 8, 12, 24, which corresponds to the mean duration of staying within each compartment 24, 12, 8, 6, 4, 3, 2 and 1 month.

In Fig. B1 are the examples of the time dependence of different compartments for *n*=3 and *n*=12:

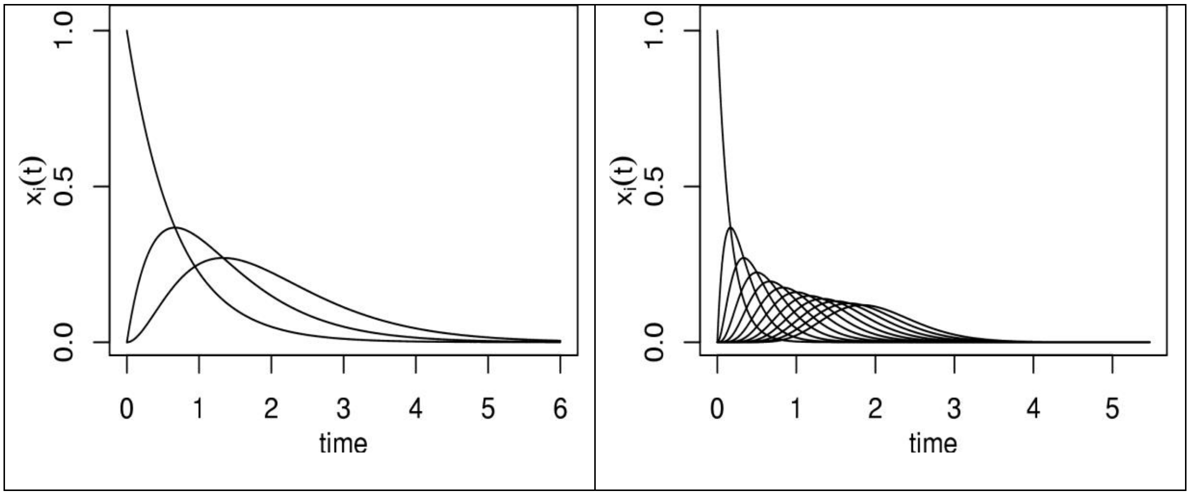

Fig. B1.

For the total population and for the hazard function the results are the following.

a) No additional mortality, m=0, everyone die immediately after leaving n-th compartment:

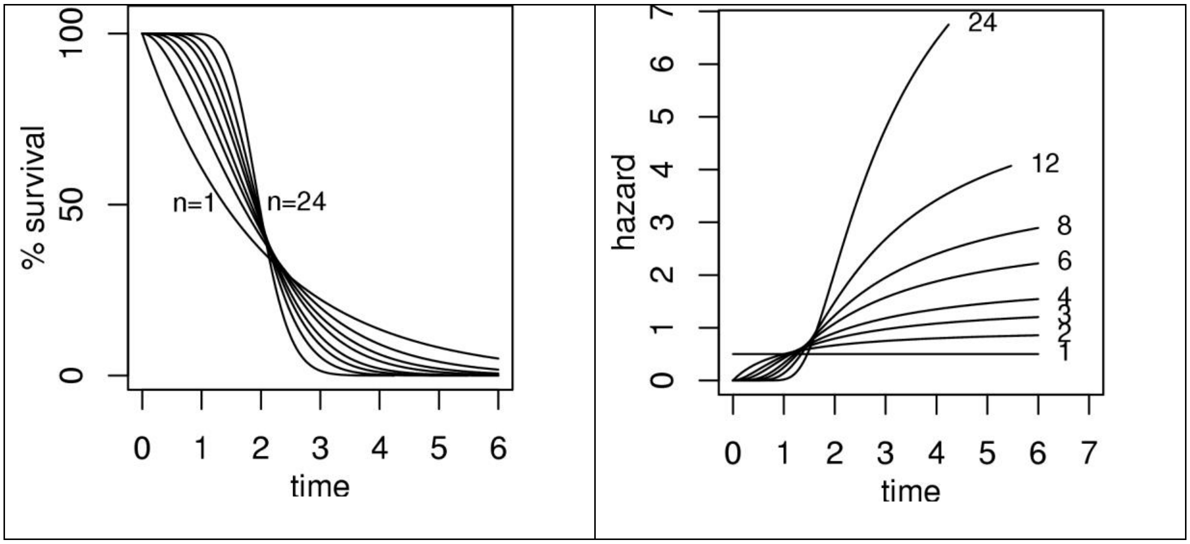

Fig. B2.

b) Additional mortality $m_i = 0.2$

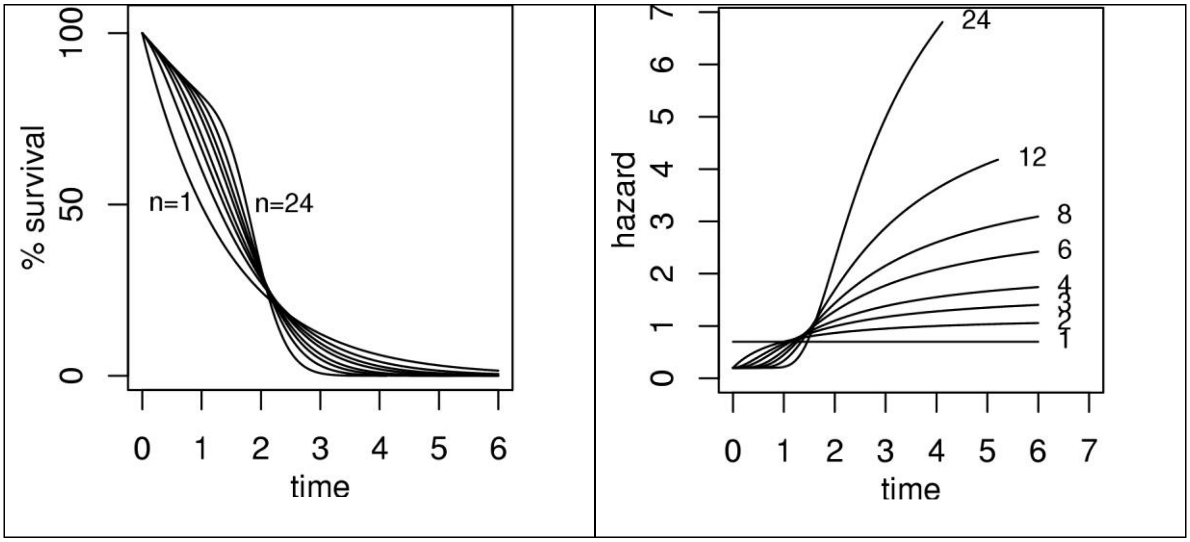

Fig. B3.

The greater is n, the closer is the survival distribution to a step-like function.

Fig. A1 in Appendix A was obtained in a similar way.
